# The temperate forest phyllosphere and rhizosphere microbiome: a case study of sugar maple

**DOI:** 10.3389/fmicb.2024.1504444

**Published:** 2025-01-15

**Authors:** Morgane Enea, Jacob Beauregard, Tonia De Bellis, Maria Faticov, Isabelle Laforest-Lapointe

**Affiliations:** ^1^Département de Biologie, Université de Sherbrooke, Sherbrooke, QC, Canada; ^2^Département de Biologie, Centre Sève, Université de Sherbrooke, Sherbrooke, QC, Canada; ^3^Centre d'Étude de la Forêt Université du Québec à Montréal, Montreal, QC, Canada; ^4^Department of Biology, Dawson College, Montreal, QC, Canada; ^5^Department of Biology, Concordia University, Montreal, QC, Canada; ^6^Quebec Centre for Biodiversity Science, Department of Biology, McGill University, Montreal, QC, Canada; ^7^Department of Ecology, Environment and Plant Sciences, Stockholm University, Stockholm, Sweden

**Keywords:** *Acer saccharum*, sugar maple, tree-microbe interactions, rhizosphere, phyllosphere, arbuscular mycorrhizal fungi, climate change

## Abstract

The interactions between sugar maple (*Acer saccharum*, Marshall) and its microbial communities are important for tree fitness, growth, and establishment. Despite recent progress in our understanding of the rhizosphere and phyllosphere microbial communities of sugar maple, many outstanding knowledge gaps remain. This review delves into the relationships between sugar maple and its microbes, as climate change alters plant species distributions. It highlights the multifaceted roles of key microbes, such as arbuscular mycorrhizal (AM) fungi and pathogens, in affecting the distribution and establishment of sugar maple in novel habitats. Furthermore, this review examines how microbial communities in different compartments contribute to tree fitness. Finally, it explores how microbial dispersal and altered species interactions under changing environmental conditions can affect sugar maple's ability to migrate beyond its current range, emphasizing the different scenarios associated with such shifts. In the rhizosphere, AM fungi are known for their roles in nutrient acquisition and improving stress tolerance. Yet, key questions remain about how these fungi interact with other microbes, how soil chemistry and climate change alter these interactions, and how the presence of beneficial microbes influences sugar maple's establishment. Additionally, the role of dark septate endophytes (DSE) in sugar maple's fitness remains underexplored, emphasizing the need for more research on their diversity and functions. In the phyllosphere, microbial communities are subject to shifts due to rising global change, with potential impacts on sugar maple's fitness. These changes may influence the tree's resistance to pathogens, tolerance to environmental stress, and overall health. Yet, our understanding of these interactions relies mostly on short-read sequencing methods targeting marker genes (e.g., 16S, ITS, 18S), which often fail to identify microbes at the species level. Limitations in molecular techniques and poor microbial reference databases hinder our ability to fully characterize tree-associated microbial diversity and functions. Future research should thus prioritize advanced molecular tools such as shotgun, hybrid, or long-read sequencing. Controlled experiments are also needed to establish causal links between sugar maple fitness and microbial communities, and to study whether microbial communities change throughout the tree's lifespan.

## 1 Introduction

As climate change is expected to accelerate over the coming decades, research suggests that the alterations of environmental conditions will affect plant species distributions, particularly in northern biomes (IPCC, 2023; Ladwig et al., 2016; Parmesan, 2006). Considerable effort is thus being put in predicting the future distribution of important plant species for ecology, economic activity, and food security to limit the negative impacts on ecosystem services and functions (Aitken et al., 2008; Rauschendorfer et al., 2022). A plant species' range is determined by multiple biotic (e.g., microbiota, herbivores) and abiotic (e.g., soil chemical profile, temperature) factors (Chase and Leibold, 2003; Pearman et al., 2008; Vandermeer, 1972). Yet, the conditions which have shaped current species ranges are quickly changing (Kellner et al., 2023; Morin et al., 2008; Savage and Vellend, 2015), forcing sessile organisms such as plants to adapt to the altered conditions of their habitat (Davis and Shaw, 2001; Moran et al., 2022; Savage and Vellend, 2015). For example, several alpine plant species are migrating to higher altitudes, as their current habitats experience an increase in mean temperature and a decrease in snow cover (Vitasse et al., 2021). Conversely, while some plant species have shifted their distribution ranges in response to current climate change, trees are not responding as quickly as predicted by distribution models (Lee-Yaw et al., 2022). Recently, several events of sudden tree mortality following heat and drought surges have been documented in ecosystems that were not predicted to be at risk, increasing the need to identify vulnerable forest components and potential ecosystem function thresholds (Hartmann et al., 2022). Therefore, achieving a better understanding of the drivers of tree species' responses to climate change is key to improving our predictions of the future of forest ecosystems.

In general, temperate tree species are following the trend of moving to higher latitudes or altitudes (e.g., to boreal regions), but this movement is slower than predicted and may not follow a straightforward northward direction (Carteron et al., 2020; Lima et al., 2024). This suggests that phenological shifts, biotic factors, or both, are slowing down migration and need to be better considered in distribution models. However, climatic conditions, including temperature, are not the sole predictors of a species' niche (Chase and Leibold, 2003; Elton, 1927; Grinnell, 1917). For tree species, their long-life expectancies, relatively short dispersal distances, and low mortality rates upon establishment are possible explanations for slow migration (Brown and Vellend, 2014; Vellend et al., 2021; Xu and Prescott, 2024). Yet, other factors such as soil properties, as well as root and soil microbes could play an important role. Studies have shown that abiotic factors such as soil pH, nutrient levels, soil structure, and porosity, as well as biotic interactions with symbiotic fungi, nitrogen-fixing bacteria, microbial pathogens, and soil mesofauna, significantly influence tree distribution and establishment (Hulshof and Spasojevic, 2020; Laughlin and Abella, 2007). In addition, climate change can create temporal and spatial mismatches between interacting species, especially if they differ in sensitivity to climatic conditions or migration rates (Gómez-Ruiz and Lacher, 2019). For example, tree migration lag is projected to correlate with a reduction of climatically compatible ectomycorrhizal fungi partners for tree species that depend on these symbioses (Van Nuland et al., 2024). Thus, the time lag between the pace of climate change and tree species migration depends on various factors, some of which are of biotic nature and warrant further research to improve our prediction capacity.

**Figure 1:**
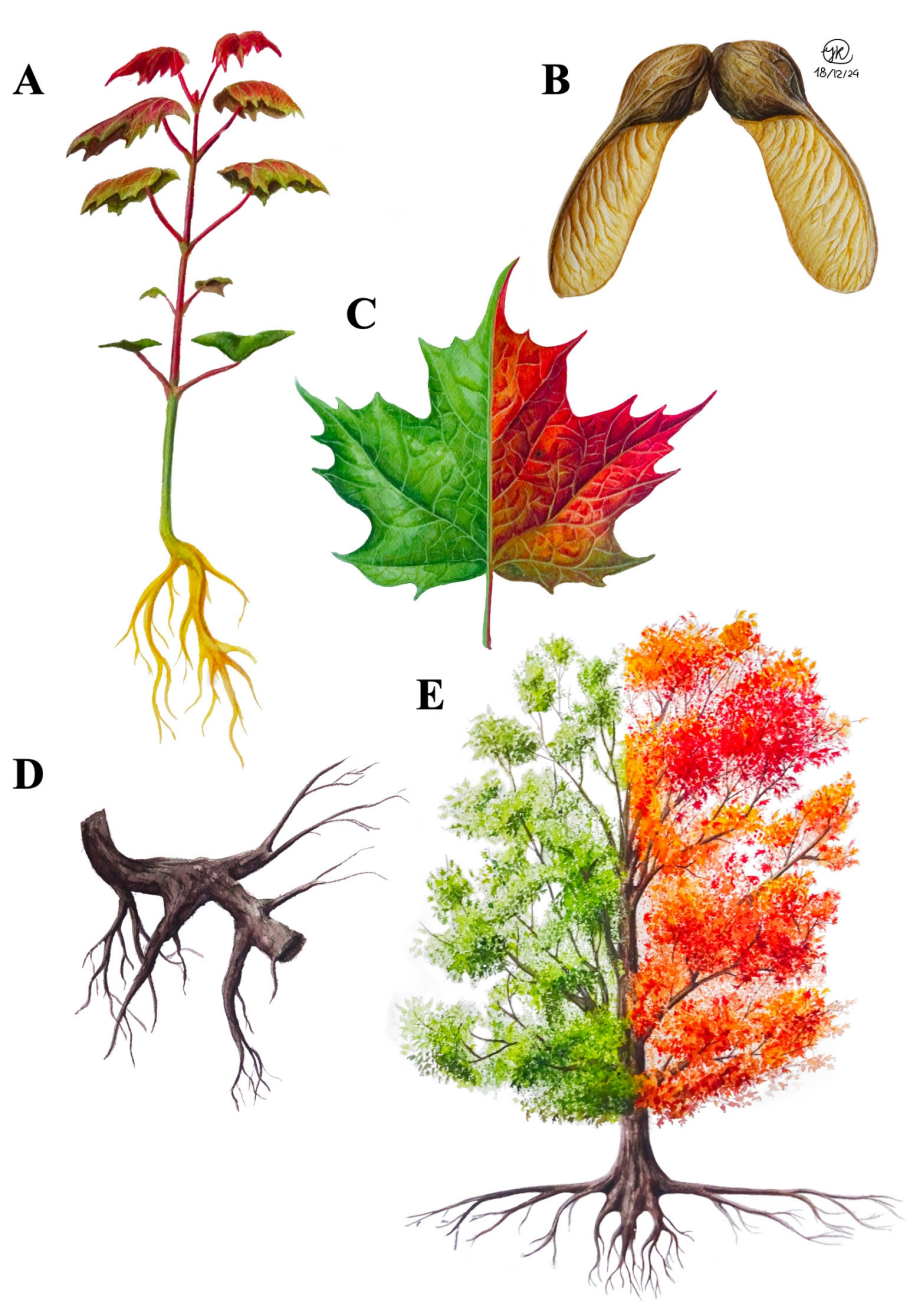
Drawings of a seedling **(A)**, samara **(B)**, mature leaf **(C)**, root section **(D)**, and adult tree **(E)** of sugar maple, created by Isabel Ramirez. The leaf drawings represent both summer and autumn seasons.

**Figure 2:**
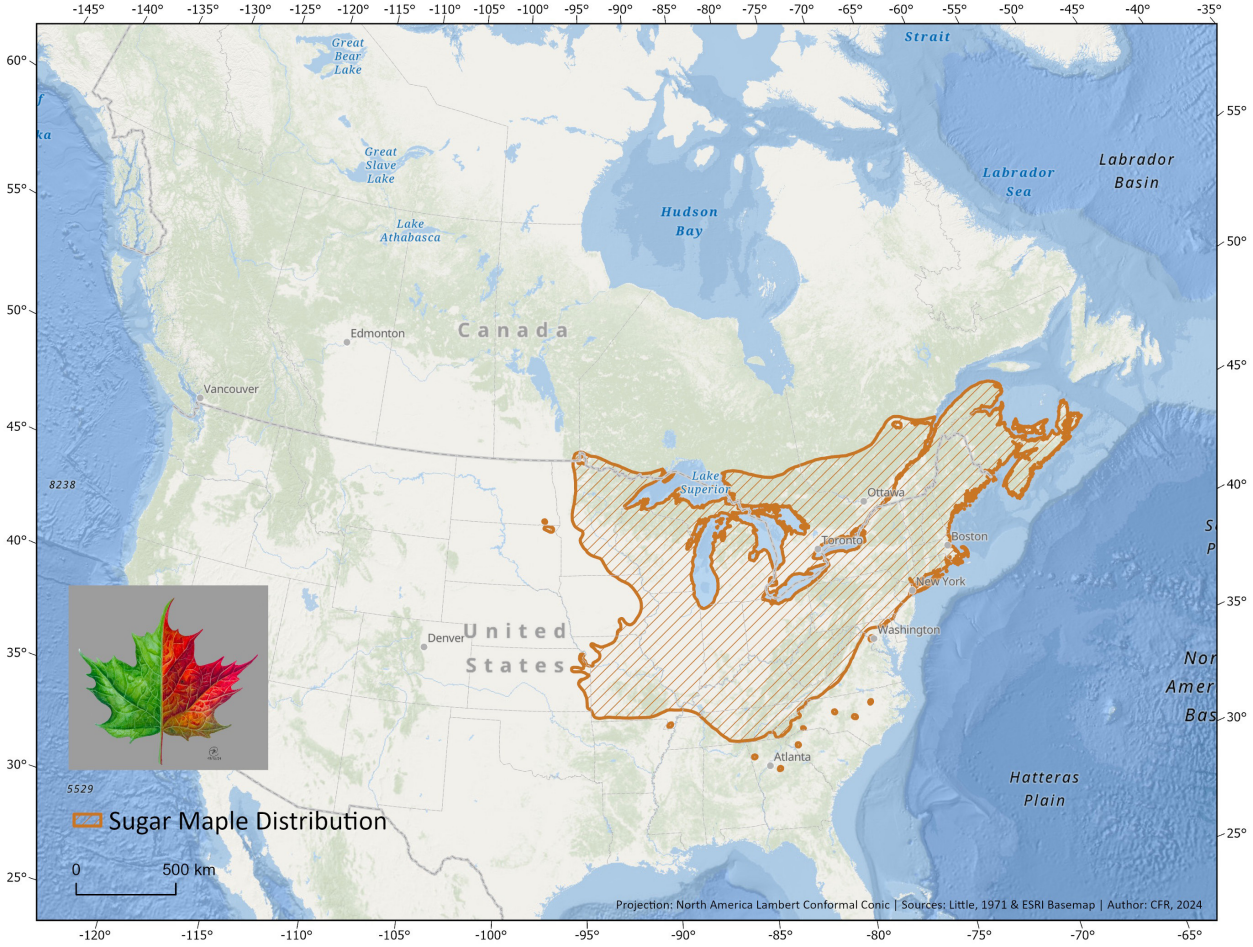
Map depicting the native range of sugar maple (*A. saccharum*). Native range data obtained from the tree species distribution range maps series in Little's “*Atlas of United States trees”* (Fryer, 2018).

In this review, we focus on the case of the sugar maple (*Acer saccharum*, Marshall, 1785; Figure 1) to summarize the current state of knowledge on tree-microbe interactions in the context of climate change and propose novel avenues of research. *Acer* L., to which the sugar maple belongs, is a key genus in broad-leaved deciduous forests of the Northern Hemisphere (Wolfe and Tanai, 1987), encompassing nearly 130 species in eastern Asia, 10 in North America, and 12 in Europe and western Asia (Gao et al., 2020). While *Acer* species likely originated in North America (Wolfe and Tanai, 1987) or in Asia (Gao et al., 2020; Li et al., 2019), the current center of *Acer* diversity is located in eastern Asia, particularly in China and Japan (Gao et al., 2020). The significantly greater species diversity in eastern Asia compared to North America is likely due to higher extinction rates and lower speciation rates in North America (Xiang et al., 2004). Maples are key components of Northern Hemisphere temperate forests, ranging from dominant canopy species such as sugar maple and red maple (*A. rubrum*) in the eastern United States and Canada, to more shrubby, sparsely distributed species in the understory such as moosewood (*A. pensylvanicum*) or riparian species such as silver maple (*A. saccharinum*). Among the diverse *Acer* species, sugar maple stands out not only for its ecological importance in North America but also for its significant cultural, economic, (Matthews and Iverson, 2017; Murphy et al., 2009), and even pharmaceutical roles (Delisle-Houde et al., 2021; Leboeuf, 2018; Maisuria et al., 2015), making it a focal point for understanding tree-microbe interactions in the context of climate change. As a dominant species of eastern North America's temperate forests, sugar maple displays a wide distribution stretching from the midwestern US to southeastern Canada (Godman, 1957; Figure 2), engendering considerable potential genetic diversity (Graignic et al., 2018). Moreover, much like the eastern white pine (*Pinus strobus*, L.), this sapindaceous species plays a keystone role: its disappearance could jeopardize the ecosystem balance and functioning, with consequences that are difficult to predict (Horsley et al., 2002; Uprety et al., 2017). While many species distribution models for trees currently lack explicit incorporation of microbial interactions (but see Van Nuland et al., 2024 and Allsup et al., 2023), understanding sugar maple's microbial symbiotic partners and antagonists is crucial for better predicting the species' future distribution shifts.

Previous studies have shown that sugar maple assembles diverse and species-specific root and leaf microbiomes, with potential impacts on tree growth, immunity, and survival. First, the origin of the soil on which sugar maple grows appears to play a key role. Despite improved regeneration under colder temperatures, which corresponds to current northern range limit climatic conditions, sugar maple survival and biomass after transplants are up to 50% higher on soil from the center of its range (Brown and Vellend, 2014; Carteron et al., 2020). This suggests that this tree species is highly dependent on root biotic interactions (e.g., in the rhizosphere with arbuscular mycorrhizae) for successful establishment in a newly available niche or in adaptation to climate change, irrespective of the physico-chemical properties of the soil (Brown and Vellend, 2014; Carteron et al., 2020; Pitel and Yanai, 2014). Second, sugar maple leaves have also been the focus of several studies on tree-microbe interactions in the phyllosphere, demonstrating that the local abiotic environment of trees drives leaf microbial colonization (Laforest-Lapointe et al., 2016a; Wallace et al., 2018). In addition to the role of leaf microbes in host immunity, these microbial communities have been associated to plant community productivity (Laforest-Lapointe et al., 2017; Li et al., 2023). While several studies have now shown that tree microbiomes play important roles for tree population dynamics and range shifts, we still ignore much of the mechanisms structuring tree-microbe interactions. Temperate forest ecosystems worldwide are currently threatened by the higher frequency of extreme weather events, but also by fires, increased drought stress, and intensified pest outbreaks (Gilliam, 2016). A different response could be expected in boreal forests, which may be linked to concomitant increases in temperature and atmospheric CO_2_. Different forest biomes will thus not respond to further global change in uniform ways (Forzieri et al., 2022). In line with these trends, sugar maple is in decline. Since the 1960s, a decrease in radial growth, crown dieback, and increased mortality have been observed in maple groves across North America, as well as symptoms linked to excessive hydric and nutritional stress and opportunistic biotic attacks (Bose et al., 2017; Horsley et al., 2002). The emblematic status of sugar maple and the stakes involved in its conservation for temperate forests in North America highlight the urgent need to build stronger scientific foundations to predict and mitigate the consequences of climate change for this tree species. Additionally, temperate forests dominated by sugar maple provide rich reservoirs of biodiversity, thus tying the conservation of this species to the fate of many other co-occurring species (i.e., umbrella species). To prevent sugar maple from declining in the face of global change, it will be crucial to gain a better understanding (1) of its capacity to shift north in pace with climatic changes and (2) of the contributions of surrounding microbial communities in this process.

The relatively recent decline in sugar maple populations, and the potential implications of global change in this phenomenon have led to several investigations of tree-microbe interactions that occur at its geographic range limit. Recent studies have shown that a shift in tree-microbe associations occurs at the edge of the sugar maple distribution range, and there is evidence for this in the microbial communities of both below- and aboveground environments (De Bellis et al., 2022; Wallace et al., 2018). This shift could have major consequences for sugar maple, as seedlings experienced reductions in regeneration when grown in soils from their range edge environments and beyond, relative to within their core distribution (Carteron et al., 2020). However, when sugar maples are transplanted to the northern edge of their range in the field, they have also been shown to benefit from the reduced pressure from natural enemies (Urli et al., 2016). This phenomenon can be explained by the enemy release hypothesis (Keane and Crawley, 2002). For example, two studies in the province of Québec (Canada) have documented a decrease in foliar damage from herbivorous insects and pathogenic fungi at sugar maple northern latitudinal and elevational limits (De Bellis et al., 2019; Urli et al., 2016). While sugar maple appears to benefit from the reduced biotic stress at the range edge, shifts in microbial community structure (i.e., which taxa are present and in what proportion) can have significant consequences. Indeed, each forest type harbors distinct microbial communities, with varying abundances of saprophytic and pathogenic taxa (Bahram et al., 2020; Heděnec et al., 2020; Netherway et al., 2021). As sugar maple migrates northward, reductions in microbial diversity—particularly the loss of key taxa such as arbuscular mycorrhizal (AM) fungi—may limit their growth and survival (Chamard et al., 2024; De Bellis et al., 2022).

Within the core of their range, sugar maple may experience negative population feedback due to the accumulation of natural enemies (Janzen, 1970; Jia et al., 2020). Yet, Bennett et al. (2017) found that sugar maple experiences lower levels of negative population feedback compared to other temperate tree species, thus providing a potential competitive advantage and facilitating their recruitment at the northern edge of their range. Even so, abiotic factors, such as colder temperatures and soil conditions (Solarik et al., 2018), along with biotic factors such as reduced microbial diversity (Wisz et al., 2013), create significant constraints on sugar maple's range shifts, which are key to understanding future changes in the temperate-boreal ecotone. With this said, the range shift of sugar maple may not occur in a strictly northward fashion, but could follow a more complex pattern involving different expansion and contraction phases, as predicted by recent studies on the range expansion of Norway maple (*A. platanoides*; Lima et al., 2024) and red maple (*A. rubrum*; Brice et al., 2019). This result brings forward several key considerations for both sugar maple, and their associated microbes. For instance, each expansion phase could involve different microbial community assemblages, influencing host tree nutrient acquisition and stress tolerance. As sugar maple encounters new environments, the species may face challenges in forming or maintaining beneficial microbial relationships, particularly if mutualistic taxa are absent (Perreault and Laforest-Lapointe, 2022). Understanding these complex patterns and tree-microbe interactions is essential for predicting sugar maple migration and adaptation, as temperatures are expected to rise in the temperate and boreal biomes, in combination with more frequent and intense droughts and wildfires.

In this review, we provide a comprehensive synthesis of the current knowledge of sugar maple rhizosphere and phyllosphere tree-microbe interactions in the context of global change. Drawing from the literature on the sugar maple microbiome, we aim to answer the overarching question: *Which roles will microbial communities play in determining sugar maple distribution beyond its current northern limit as climate change accelerates?* We focus on how mutualistic microbes, such as AM fungi, and pathogens influence sugar maple's response to climate change and its potential for range expansion. Additionally, we explore the roles of microbes across different plant compartments—roots and leaves—and how they affect the tree's performance and adaptation. Finally, we examine how microbial dispersal and shifting species interactions under climate change may shape the tree's ability to migrate beyond its current range.

## 2 Tree-microbe interactions

Studying trees is neither fast, cheap, nor simple. In comparison with the plant model study system *Arabidopsis thaliana*, trees grow slower, have larger genomes, and are thus more difficult to manipulate genetically (Arnold et al., 2024; Clark, 1949; Vacher et al., 2016). It is therefore easy to grasp the challenges of working with trees as a study system, which has henceforth led to a lack of studies on tree-microbe interactions. Yet, through their leaves, trunks, branches, and roots, from the small seedling to the mature tree, these plants provide a wide variety of habitats for microorganisms, as well as a large surface area for exchanges (Clark, 1949; Perreault and Laforest-Lapointe, 2022; Vacher et al., 2016). This makes the study of tree microbiota particularly complex, but just as exciting. Complex systems are governed by a set of abiotic and biotic forces and their interactions, as well as by stochastic events, leading to the establishment of a multi-faceted dynamic. Tree-microbe interactions form a complex system, which thus requires multidisciplinary research initiatives to continue to push the field forward.

In the preceding decade, several studies leveraging next-generation sequencing have shown that sugar maple microbial communities are exceedingly diverse, encompassing a wide range of bacteria, fungi, archaea, viruses, and other microorganisms (Tables 1, 2). The study of Wallace et al. (2018) demonstrated that sugar maple bacterial communities, across all compartments (inside and outside roots and leaves), consisted of four primary phyla and 11 major classes. Among these, Proteobacteria accounted for 59.4% of the sequences and included four classes: Alpha-, Beta-, Delta-, and Gamma-proteobacteria. As for fungi, this study showed that the dominant fungal phyla were Zygomycota, Ascomycota, and Basidiomycota, while the most abundant classes included Dothideomycetes, Sordariomycetes, and Agaricomycetes. To our knowledge, there are no studies focusing on protists or archaea in the sugar maple microbiome yet, while there is one study that reported the presence of methanogenic archaea in the bark of yellow-paint maple, which is native to Asia (*A. pictum;* Harada et al., 2024). Similarly, only a handful of studies have focused on viral members of sugar maple microbial communities. These studies were focused on viruses that cause diseases accompanied by chlorotic spots and mottle symptoms (Lana et al., 1980; Rumbou et al., 2021). For example, Rumbou et al. (2021) employed RNA-Seq technology to study the viral agents of maple trees in urban parks, demonstrating the presence of mottle-associated virus (MaMaV). Although there has been remarkable progress in our understanding of sugar maple bacterial and fungal microbial communities, the limited research on archaea, viruses, and protists highlights the need for future studies to uncover their roles, interactions, and impacts on tree fitness and range expansion, particularly in the context of global change.

**Table 1:** A list of studies that explored the interactions between sugar maple and microbial communities belowground.

**Reference**	**Location**	**Microorganisms**	**Compartment**	**Methods**
De Bellis et al. (2002)	Mixed deciduous forests at Réserve faunique de Portneuf and Station Forestière in Duchesnay (QC, Canada)	AM and EM fungi	Roots	Microscopy, ECM morphotyping
Klironomos (1995)	Southern to northern Ontario (ON, Canada)	AM fungi	Roots and soil	Microscopy and spore collection from soil
Klironomos et al. (1993)	Maple forests in Waterloo (ON, Canada) and St. Lawrence floodplain (QC, Canada)	AM fungi	Roots and soil	Microscopy and spore collection from soil
De Bellis et al. (2019)	Sugar and Norway maples at Morgan Arboretum (QC, Canada)	Fungi and bacteria	Roots and rhizosphere	Metabarcoding and microscopy
Coughlan et al. (2000)	Healthy and declining sugar maple forests at Lac Clair and Portneuf (QC, Canada)	AM fungi	Roots and soil	Microscopy, spore collection from soil
Cooke et al. (1992)	Biological Station of U. de Montréal (QC, Canada)	AM fungi	Roots	Microscopy
Cooke et al. (1993)	Biological Station of U. de Montréal (QC, Canada)	AM fungi	Roots	Microscopy
Chamard et al. (2024)	Elevational gradients at Parc National du Mont Mégantic and Réserve naturelle des montagnes vertes (QC, Canada)	Bacteria, fungi, and AM fungi	Roots and rhizosphere	Microscopy and metabarcoding
Carteron et al. (2020)	Parc national du Mont-Mégantic (QC, Canada)	Endophytes and AM fungi	Roots and soil	Greenhouse experiment, microscopy

**Table 2:** A list of studies that explored the interactions between sugar maple and microbial communities aboveground.

**Reference**	**Location**	**Microorganisms**	**Compartment**	**Methods**
De Bellis et al. (2022)	Latitudinal gradient in Québec (QC, Canada) RESEF (Le Réseau d'Étude et de Surveillance des Écosystèmes Forestiers Québécois)	Bacteria, fungi, and AM fungi	Rhizosphere, roots, and leaves	Metabarcoding
Wallace et al. (2018)	Elevational gradient at Parc National du Mont-Mégantic (QC, Canada)	Bacteria and fungi (epi- and endophytes)	Leaves and roots	Metabarcoding
Laforest-Lapointe et al. (2016a,b)	Four natural temperate forest stands in Québec: Sutton, Abitibi, Gatineau, and Bic (QC, Canada)	Bacteria (epiphytes)	Leaves	Metabarcoding
Laforest-Lapointe et al. (2017)	Common garden experiment (IDENT), Ste-Anne-de-Bellevue (QC, Canada)	Bacteria (epiphytes)	Leaves	Metabarcoding
Demarquest and Lajoie (2023)	Nine forested sites across eastern Ontario (ON, Canada), north-eastern USA, and Québec (QC, Canada)	Bacteria (epiphytes and endophytes)	Leaves	Metabarcoding
N'guyen et al. (2022)	Sugar maple sap producers in Québec (QC, Canada) and New Brunswick (NB, Canada)	Fungi and bacteria	Sap	Metabarcoding

The microbial communities associated with sugar maple colonize different plant compartments, which offer unique microhabitats that support different microbial assemblages (De Bellis et al., 2022; Laforest-Lapointe et al., 2016a,b; Wallace et al., 2018). The soil surrounding the roots, or the rhizosphere *sensu stricto*, is one of the most dynamic environments for microbial activity and interactions (Berendsen et al., 2012). In sugar maple, the rhizosphere is teeming with bacteria and fungi (Chamard et al., 2024; Wallace et al., 2018) that contribute to nutrient cycling, organic matter decomposition, and protection against soil-borne pathogens (Berendsen et al., 2012; Mohanram and Kumar, 2019; Raaijmakers et al., 2009). Sugar maple roots (Figure 1D) are colonized by both endophytic (i.e., inside plant tissues) and epiphytic (i.e., on plant surfaces) microbes. Mycorrhizal associations, particularly with arbuscular mycorrhizal (AM) fungi, are prominent in this compartment, enhancing the tree's nutrient uptake and defenses (Begum et al., 2019; Netherway et al., 2021; van der Heijden et al., 2008). However, the relationship between AM fungi and the conspecific density of trees forming such associations presents a more nuanced picture. Research suggests that tree species associated with AM fungi can experience relatively strong negative population feedbacks, leading to a reduction in the density of juvenile stems in proximity to adult trees (Bennett et al., 2017; Delavaux et al., 2023). This negative density-dependent effect is thought to be attributed to both general competitive stand dynamics, wherein adult trees monopolize light and nutrient resources to the detriment of juvenile seedlings, and to the accumulation of species-specific pathogens that make it difficult for young trees to establish (Connell, 1971; Delavaux et al., 2023; Janzen, 1970). Overall, the impact of AM fungi on sugar maple likely depends on myriad factors, including local environmental conditions and competition with other species, making the outcome of maple-AM fungal interactions highly context-dependent. The subsection below entitled “Rhizosphere” summarizes the current state of knowledge on sugar maple root-microbe interactions (Table 1).

The aerial surfaces of plants, or the phyllosphere *sensu lato*, also provides an important habitat for microbes. Common sugar maple leaf microbial colonists include bacteria from Alpha- and Gamma-proteobacteria, Hymenobacteraceae, Beijerinckiaceae, Pseudomonadaceae classes as well as fungi from Dothideomycetes, Eurotiomycetes, Leotiomycetes, and Sordariomycetes classes (Wallace et al., 2018; Laforest-Lapointe et al., 2016b). These microbes can notably contribute to tree host fitness by outcompeting leaf pathogens or enhancing photosynthetic efficiency (Bamisile et al., 2018; Khare et al., 2018; Vacher et al., 2016). Nevertheless, evidence of mutualism in endophyte-tree symbioses has often been inconclusive (Sieber, 2007). It is however likely that plants would struggle to endure many environmental stresses without these associations, as is evident from host-microbe interactions belowground. Yet, there is little evidence of biocontrol potential of foliar endophytes colonizing sugar maple in nature (but see Pehl and Butin, 1994 Sieber and Dorworth, 1994). Moreover, several studies have also demonstrated no differences in fungal and bacterial endophyte community composition and diversity between phytopathogen-infected and asymptomatic *A. campestre* and *A. platanoides* leaves (Wemheuer et al., 2019), two closely-related maple species. The similarity between infected and asymptomatic leaves could be explained by the fact that some pathogens responsible for the tree diseases are present in asymptomatic leaves as latent infections (Abdelfattah et al., 2017; Cross et al., 2017). The subsection below entitled “Phyllosphere” summarizes the current state of knowledge on sugar maple leaf-microbe interactions (Table 2).

Even if cross-sectional studies have largely dominated the research into microbiomes, the fluctuating dynamics of the growing season in temperate forests (from bud burst to leaf senescence) has oriented many researchers toward temporality. Thus, early research on sugar maple bacterial and fungal temporal dynamics primarily focused on understanding how microbial community structure and alpha diversity changed over the seasons. For roots, with a few exceptions, there is a lack of studies exploring microbial community temporal dynamics. In a study led by De Bellis et al. (2019), fungal community composition remained unaltered throughout the growing season (from May to October), while bacterial community composition showed significant changes. Other studies mainly explored temporal variation in AM fungal abundance. For example, Cooke et al. (1992) observed seasonal variation in the incidence of AM fungi, but these differences were not consistent among years. In another study on sugar maples, Klironomos et al. (1993) found that AM colonization and hyphal length peaked in forests during spring and autumn, while spore densities were highest in autumn and decreased throughout the year. For leaves, Laforest-Lapointe et al. (2016a) sampled the foliar bacterial community of five temperate tree species, including sugar maple, at three time points during the growing season and across four sites in the Province of Québec. This study demonstrated that season had only a minor effect on bacterial community composition when compared to host species and sites. While a few studies have already explored the seasonal dynamics of microbial communities associated with sugar maple, especially aboveground, there remains a significant gap in our understanding of the temporal shifts and interactions of microbial communities, particularly in relation to AM fungi and other root-associated microbes.

The roots and leaves are not the sole vessels of microbial life in sugar maple trees. First, the well sought-after maple sap has been shown to house a diverse suite of bacterial strains from the phyla Firmicutes, Actinobacteria, and Proteobacteria, as well as multiple fungal genera such as *Rhodosporidiobolus, Cyberlindnera, Curvibasidium, Cystofilobasidium, Itersonilia, Phenoliferia, Phaffia*, and *Vishniacozyma* (N'guyen et al., 2022). Additionally, Filteau et al. (2010) showed that the microbial communities of sugar maple sap change during sap production in spring, with *Pseudomonas* and *Rahnella* being the two most represented bacterial genera. Moreover, sugar maple seeds could also be an important reservoir for diverse microbial members. While there is yet no work characterizing sugar maple seed microbial communities, several studies have demonstrated that microbes in tree seeds have co-evolved with their host tree species and provide special growth traits for tree survival (Abdelfattah et al., 2023, 2021; War et al., 2023). Clearly, there is a contribution of microbial members from the seed to the root and leaf compartments (Abdelfattah et al., 2021; Faticov et al., 2023). In view of these results, prominent knowledge gaps remain: (1) How do these tree-microbe exchanges occur?; (2) Which factors regulate them?; and (3) What are their impacts on tree performance?

To address these questions, it is important to draw on the foundations of community ecology which aims to understand which abiotic and biotic forces determine the structure and dynamics of communities at different spatial and temporal scales. The evolution of community ecology in the 20th century has laid a foundation for studying assembly processes that govern not only communities of macroorganisms, but also of microorganisms. Clements' early view of communities as “superorganisms” suggested that species are assembled in non-random combinations (Clements, 1916), while Gleason emphasized the role of climatic variability in shaping species responses and community structure (Gleason, 1939). Niche theory, articulated by Grinnell and Elton and later refined by Hutchinson to include fundamental and realized niches (Elton, 1927; Grinnell, 1917; Hutchinson, 1959), stands in contrast to Hubbell's Neutral Theory, which argues that communities are shaped by random processes rather than organismal traits (Hubbell, 2001). According to Vellend (2010), four primary processes—selection, drift, dispersal, and speciation—govern community assembly. While these concepts have shaped ecological thought, their application to microbial communities presents unique challenges due to the small size, abundance, and rapid generation times of microbes. For instance, selection may be affected by dormant states, and drift typically occurs under conditions of weak selection and low population sizes (Cordovez et al., 2019). Ultimately, integrating these ecological principles will enhance our understanding of sugar maple-microbe interactions and their implications for forest health and tree migration.

At the core of these interactions lies the enigma of microbial dispersal. Microbial dispersal occurs through several pathways, but dispersal modes differ between below- and aboveground microbes. In the belowground world, root exudates attract and support several beneficial microbes, such as mycorrhizal fungi and rhizobacteria, which can then disperse through the soil to colonize nearby roots (Badri et al., 2009; van der Heijden et al., 2015). Soil movement, often caused by earthworms (exotic invasive species in the maple groves of Québec) or insects, transports soil particles along with associated microbes closer to the trees' roots, thus enhancing microbial colonization (Edwards and Arancon, 2022). Additionally, mycorrhizal fungi can form extensive hyphal mats and networks that cover long distances and have been shown to connect roots from different plants in specific experimental contexts (Cahanovitc et al., 2022; Figueiredo et al., 2021; Newman, 1988; Teste and Simard, 2008). This phenomenon adds another potential mechanism for microbial dispersal across temperate forests belowground (see the *Microbe-microbe interactions in the hyphosphere* subsection below; Emmett et al., 2021; Sangwan and Prasanna, 2022). However, the relevance and ecological significance of common mycorrhizal networks (CMNs) remains contentious (Table 3), as there is a lack of empirical evidence supporting their widespread occurrence and functional roles in nutrient transfer among plants (Karst et al., 2023; Walder et al., 2012; Henriksson et al., 2023; Robinson et al., 2024). This highlights the need for further investigation into the potential impact of CMNs, as well as the relevance of root and soil transfer pathways for microbial dispersal and nutrient exchange among sugar maples (Table 3).

**Table 3:** Common mycorrhizal networks and sugar maple-AM fungal interactions: key insights and future research questions.

**Topic**	**Key points on CMNs**	**Implications for sugar maple-AM fungi interactions and potential research questions**
Relevance of common mycorrhizal networks	Karst et al. (2023) challenges the ubiquity of CMNs and their ecological significance	AM fungi form associations with sugar maple roots, but do they form CMNs (if they exist) in mixed forests? If so, are there important conmycorrhizal plant partners for maple in this dynamic? Are arbuscular CMNs present in sugar maple-dominated forests? Are roots connected by the same genet? If yes, how prevalent are they?
Nutrient and resource sharing/partitioning	The extent of resource transfer through CMNs between plants is debated	Do sugar maples receive or give resources through AM fungal networks, especially in mixed-species forests? How do mixed stands with differing mycorrhizal types vary in productivity when compared to sugar maple dominant stands? What is the relevance of the CMN, root, and soil transfer pathways for nutrient exchange in sugar maple tree communities? How do AM-sugar maple interactions influence nutrient uptake under competition or stresses?
Impact on plant communities	CMNs may influence seedling establishment, species coexistence, and competition, but evidence is mixed	Do AM fungal interactions affect sugar maple seedling establishment and survival beyond its current range? Do mature maples facilitate or stymie the regeneration of seedlings, and how do AM fungal interactions influence this? Do sugar maple seedling growth or performance improve in the environment where CMNs have the potential to form or in the environments where roots are interacting? Can associations with AM fungi improve sugar maple adaptation in a changing climate?
Impact of climate change	CMNs role in buffering or exacerbating the effects of climate change on forests	AM fungal interactions may help sugar maples adapt to or mitigate climate change effects (e.g., drought, temperature fluctuations). Does climate change alter AM associations, thereby affecting sugar maple distribution?
Network stability and disruption	The stability of mycorrhizal networks is questioned, especially when ecosystems are disturbed (e.g., logging, fire, land use change). Also, the relative stability of AM vs. EM fungal networks requires further investigation.	Investigating how disturbances affect sugar maple-AM networks may provide insight into the adaptation of sugar maples to the changing environment. How resilient are sugar maple-AM networks to biotic, abiotic, and/or anthropogenic changes?
Role in carbon sequestration	The role of mycorrhizal networks in carbon cycling and sequestration. For example, evidence from a few studies suggest that mycorrhizal fungi can influence soil carbon storage and decomposition processes (Carteron et al., 2022; Choreño-Parra and Treseder, 2024; Hawkins et al., 2023)	How do sugar maple-AM interactions affect carbon storage in temperate forest soils? Do changes in AM fungal networks alter the carbon balance in these temperate forests?

In the aboveground world, microbial dispersal mechanisms are also diverse. When tree leaves drop and decompose, nutrients and some microbes residing on and within leaf tissues, such as fungi and bacteria, disperse through the decaying leaf litter, influencing the microbial community in the surrounding soil (Baldrian, 2017; Tedersoo et al., 2020). Wind, rain, and large-scale atmospheric movements also play key roles in dispersal at various geographic and temporal scales (Barbour et al., 2023; Chaudhary et al., 2020; Morris et al., 2023). The spores from leaf-colonizing fungi or bacteria can be carried away, thus inoculating the host tree crown or the neighboring trees and directly contributing to microbial dispersal (Barbour et al., 2023; Choudoir and DeAngelis, 2022). In addition, insects which feed on leaves (such as aphids, beetles, and moths) can also carry microbial pathogens or beneficial microbes on their bodies or within their guts, thus dispersing these microorganisms across leaves, trees, and forests (Coolen et al., 2022). Finally, microorganisms can also disperse through the xylem from roots to leaves and vice versa (Frank et al., 2017). Despite these insights, significant gaps remain in our understanding of how the heightened pressures of global change will affect microbial dispersal. For example, we ignore how local and global climatic shifts will influence belowground (e.g., root exudates, mycorrhizal networks, invasive species incursions) and aboveground dispersal processes (e.g., changes in precipitation patterns) within and among trees. In summary, by integrating models that forecast suitable habitats with studies on microbial interactions, researchers can better understand how microorganisms influence tree survival and growth.

## 3 Rhizosphere

Soil represents one of the most diverse ecosystems globally, thought to house over 50% of species on Earth (Anthony et al., 2023; Tedersoo et al., 2014). Soils are highly heterogeneous environments, shaped by a complex array of processes that influence plant communities, biogeochemical cycles, and both macro- and microscopic communities (Hillel and Hatfield, 2005). Trees, as long-lived and sessile organisms, play a crucial role in linking the belowground and aboveground environments. The rhizosphere, the narrow zone of soil surrounding and directly influenced by plant roots (Asiegbu and Kovalchuk, 2021; Cordovez et al., 2019), is particularly rich in biological activity due to the “rhizosphere effect”, wherein root exudates stimulate microbial activity, select for specific microorganisms, and alter soil chemistry (Berendsen et al., 2012; López et al., 2023; Prescott and Grayston, 2023). The composition of rhizosphere microbial communities is influenced by factors such as plant host identity (Quiza et al., 2023), local site conditions (Wallace et al., 2018), forest type (De Bellis et al., 2019), and edaphic and climatic factors such as pH, nutrient levels, soil temperature, and moisture (Asiegbu and Kovalchuk, 2021; Chamard et al., 2024; Rousk et al., 2010). Several studies have demonstrated an overlap in microbial taxa of the phyllosphere and rhizosphere, which was suggested to represent a “core microbiome” (Wallace et al., 2018). However, it remains difficult to identify a core microbiome, given that many studies have targeted similar age classes (e.g., tree seedlings) and have often used different molecular techniques (including different DNA extraction kits and primer pairs), when characterizing microbial members of the phyllosphere and rhizosphere. Notwithstanding recent advances, many gaps persist in our understanding of the forces governing plant rhizosphere microbial community assembly processes, particularly in how these processes affect the plant host.

While the field has provided several studies on the impacts of climate change on the persistence of sugar maple populations (Carteron et al., 2020; Collin et al., 2018; Solarik et al., 2018), it is not clear how microbial interactions in the rhizosphere will contribute to sugar maple establishment in new environments beyond its core range. Nevertheless, these studies have laid a strong foundation for future research to explore the sugar maple root microbiome and its responses to a changing climate.

### 3.1 Rhizosphere bacteria: key drivers and ecology

The key factors influencing bacterial community assembly in sugar maple roots and in the rhizosphere include local habitat configuration (e.g., elevation), forest type (e.g., temperate *vs*. boreal), host identity, climatic and soil variables such as temperature, moisture, soil pH, as well as nutrient content (Chamard et al., 2024; De Bellis et al., 2019; Wallace et al., 2018). Many bacterial phyla play crucial roles for their tree hosts, interacting within the rhizosphere in ways that can significantly impact the functioning of plant roots (Asiegbu and Kovalchuk, 2021; Cordovez et al., 2019). In spite of the variations in bacterial communities due to aforementioned factors, Wallace et al. (2018) suggested the existence of a core microbiome for sugar maple, comprising a shared set of microorganisms across different habitats and plant compartments. Interestingly, several studies on the bacterial communities of sugar maple, including those of De Bellis et al. (2019, 2022), emphasize similar bacterial taxa across different sites and forest types, highlighting a potential role in tree fitness outcomes. Among important taxa are the four classes Alpha-, Beta-, Delta-, and Gamma-proteobacteria. While these results concur at a coarse level of taxonomic resolution (limited by the resolution of 16S rRNA marker gene sequencing), these classes do contain many important taxa known for their impacts on tree performance and immunity (Asiegbu and Kovalchuk, 2021; Raaijmakers et al., 2009). Further research is warranted to investigate the species- and strain-level diversity and functional profiles of these bacterial communities as well as their roles for sugar maple performance.

**Figure 3:**
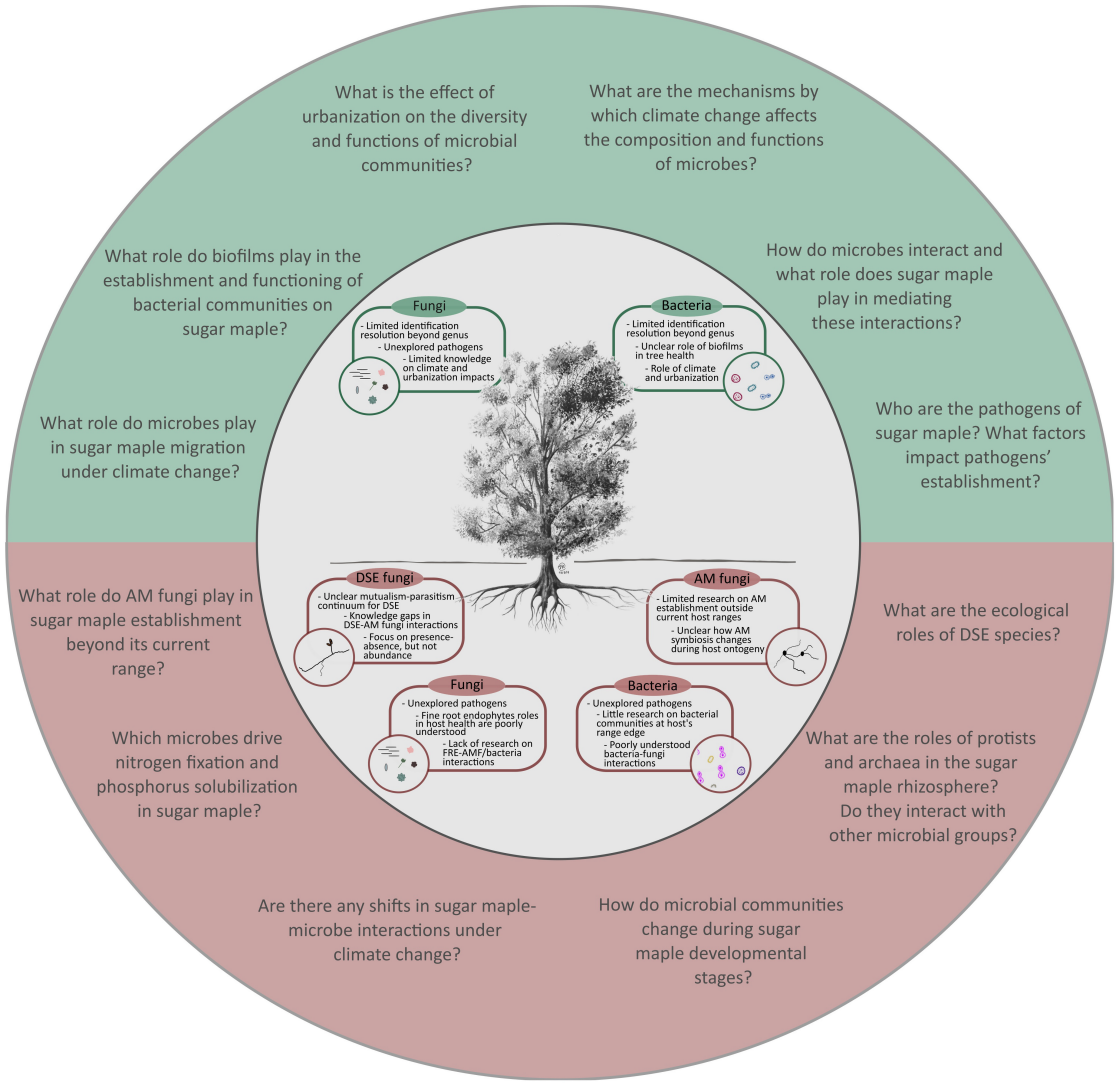
Schematic overview of the main knowledge gaps and the relevant questions in the research on sugar maple-microbe interactions. Knowledge gaps are presented separately for below- (red) and aboveground (green) microbial groups (inner circle with a tree). Relevant questions (outer circle) are proposed to advance research on sugar maple-microbe associations.

Beyond root bacterial community composition, shifts in bacterial alpha-diversity across sites and plant host compartments were also detected. De Bellis et al. (2022) found that forest type (i.e., temperate, mixed, or boreal) had a significant impact on root bacterial Shannon diversity, where diversity decreased from temperate to boreal forests. Similarly, Wallace et al. (2018) and Chamard et al. (2024) found decreases in bacterial Shannon diversity along several elevation gradients. Of note, Chamard et al. (2024) also identified a parabolic relationship between altitude and alpha diversity at one site, indicating that, in some locations, bacterial diversity may increase at intermediate elevations before declining at higher altitudes. In the study by Wallace et al. (2018), sugar maple seedlings growing at their range edge had distinct bacterial communities compared to those in the core distribution range, with root bacterial diversity being lower at the edge of the tree's distribution. Overall, sugar maples are likely to encounter distinct microbial communities at the edge of their range compared to the core, which could challenge the species' establishment in new habitats. Understanding the ecological roles of the microbial members is thus essential. Future research should thus focus on elucidating sugar maple bacterial communities at a finer scale and in different environmental contexts (e.g., different soil conditions, dominant tree communities, temperature/humidity regimes) to determine (Figure 3) (1) how these communities may shift; (2) who are the key players in this process; and (3) what role they play in the rhizosphere of sugar maple.

### 3.2 Rhizosphere fungi: key drivers and ecology

The limited studies on the root fungal communities of sugar maple reveal significant overlap, along with some intriguing discrepancies. For example, while De Bellis et al. (2019) largely agreed with Wallace et al. (2018) regarding the dominant bacterial phyla associated with sugar maple roots, their findings on fungi showed notable differences. De Bellis et al. (2019) identified Ascomycota and Basidomycota as the most abundant fungal phyla, both of which are common for forest soils. In contrast, Wallace et al. (2018) found that the phylum Zygomycota predominated, followed by the Ascomycota. Although the differences in sampling sites and timing could explain some of this variation, it may be also explained by the choice of the compartment, where Wallace et al. (2018) focused on the rhizosphere and roots, while De Bellis et al. (2019) focused exclusively on roots. It is also important to note that the Zygomycota phylum has since been reclassified into two distinct phyla: Mucoromycota and Zoopagomycota (Spatafora et al., 2016). Zoopagomycota primarily includes parasites and pathogens of small animals, whereas Mucoromycota encompasses subphyla such as Mortierellomycotina, Mucoromycotina, and Glomeromycotina, which include several taxa known to form positive associations with roots (although Glomeromycotina is often used interchangeably with Glomeromycota, which is often still considered as a distinct phylum). These associations can take the form of mycorrhizae, mycorrhiza-like relationships, and root endophytes (Bonfante et al., 2020; Orchard et al., 2017; Spatafora et al., 2016). In addition to the AM fungi (i.e., Glomeromycota) which warrant a separate discussion, a widespread group of beneficial root-fungal symbionts within the Mucoromycotina known as “Fine Root Endophytes” (FRE), have the ability to establish mycorrhizal-like associations with vascular plants (Hoysted et al., 2019). Although their ecological significance is not yet fully understood and their symbiotic functions have only recently been recognized, FRE have been identified as important sources of phosphorus and nitrogen for their host plants (Hoysted et al., 2023). Given their widespread occurrence, tendency to co-occur with AM fungi, and potential contributions to plant nutrition and fitness, further research is needed to explore the role of this fungal group and other root associated fungi, particularly in relation to sugar maple.

Another intriguing group of fungi often associated with sugar maple roots is the dark septate endophytes (DSE). Very few studies have looked at their relationship with sugar maple (but see Chamard et al., 2024 and De Bellis et al., 2019), and have largely focused on presence/absence data. DSE are a ubiquitous group of ascomycetes fungi found in association with plant roots worldwide (Newsham, 2011). DSE have been shown to span the mutualism-parasitism continuum (Grünig et al., 2008; Jumpponen, 2001; Mandyam and Jumpponen, 2014), acting as a biocontrol against fungal pathogens in some systems (Wang et al., 2022) and as growth promoting fungi in others (Liu et al., 2022; Santos et al., 2021). Additionally, they have been shown to act parasitically or have neutral impacts on plant growth and survival outcomes (Mayerhofer et al., 2013), further obscuring our understanding of their ecological roles. Netherway et al. (2024) demonstrated that DSE associations are widespread in European forests and may play a key role in shaping root and soil microbiomes of different tree species. Supporting this, Chamard et al. (2024) found that DSE colonization showed a significantly associated with root and soil AM fungi of sugar maple. Furthermore, in a study comparing co-occurring maple species, De Bellis et al. (2019) reported higher DSE colonization rates in sugar maple compared to Norway maple, with DSE being more abundant in sugar maple roots. In the same study, the order Helotiales, within which many DSE belong, was found to be one of the most dominant fungal orders associating with sugar maple roots. While the ecological significance of DSE-tree interactions remains unclear, evidence suggests that DSE could play an important role for sugar maple growth by shaping their root-associated microbiomes and altering growth outcomes. It is, however, likely that the wide range of observed ecological functions and tree-DSE interaction outcomes is due to the distribution of DSE taxa across several distinct orders of the phylum Ascomycota (Watkinson et al., 2015). Given the high prevalence of DSE in maple roots, characterizing key DSE taxa and functions will be important, especially as sugar maple is projected to migrate to higher elevations and boreal zones where these fungi are thought to be more abundant (Figure 3).

Despite the ecological and economic significance of sugar maple, research on its root pathogens has been limited, with studies of mortality agents focusing mainly on insect herbivory (Hakimara and Despland, 2023; Horsley et al., 2002). Some studies have investigated root pathogens and their interactions with defoliation and herbivory in sugar maple (Bauce and Allen, 1992). *Armillaria*, a genus containing notable fungal pathogens affecting sugar maple roots, varies in both host preferences and pathogenicity—a few species can cause rapid mortality, while others merely weaken the host (Horsley et al., 2002; Morrison et al., 1985). The impact of *Armillaria* species often act synergistically with attacks by boring beetles or via herbivory, with the aforementioned stressors weakening the tree host and facilitating infection as well as the subsequent mortality of sugar maple (Bauce and Allen, 1992; Horsley et al., 2002). In view of the broad distribution of *Armillaria* spp. in sugar maple's range, identifying the species that target this tree, and its synergisms with other disturbance agents, could be key for effective management of these widespread and often virulent pathogens.

### 3.3 Arbuscular mycorrhizal fungi: ecology and interactions with sugar maple

One of the most striking features of the sugar maple is that it is among the few dominant northern temperate tree species that exclusively associates with AM fungi (Cooke et al., 1992; Ouimet et al., 1996). Most tree species in higher latitudes, such as beech, birch, and several coniferous species, typically associate with ectomycorrhizal (EM) fungi (Soka and Ritchie, 2014). AM-dominated forests have been shown to exhibit significant differences in carbon storage capacity, C/N ratios, nutrient cycling rates, and microbial communities relative to those dominated by EM species (Averill et al., 2018, 2021; Eagar et al., 2023; Kadowaki et al., 2018). As global climates shift, the suitable habitats for EM plants and fungi are expected to shrink (Van Nuland et al., 2024). This could lead to a competitive advantage for sugar maple in northern forests (Boisvert-Marsh et al., 2022; Brice et al., 2020), where increasing forest “arbuscularization” (i.e., a process of increasing AM species relative to EM tree basal area occurring across much of North America; Averill et al., 2018; Jo et al., 2019) can reshape ecosystem dynamics, potentially favoring AM-associated trees. In addition, given the global distribution of AM fungi and their low host specificity, it is often assumed that trees associating with AM fungi, such as sugar maple, will face fewer challenges when colonizing new areas outside of their native ranges, due to a ready access of symbiotic partners (Dickie et al., 2017). While studies have explored the differences in the AM fungi of sugar maple and the naturalized Norway maple within their core range (De Bellis et al., 2019), the potential for symbiont aided host-expansion facilitation has largely focused on EM fungi and trees (Van Nuland et al., 2024). Further research is thus needed to assess how AM-associated trees, particularly sugar maple, will adapt to novel environments—such as transitioning into boreal regions—where the availability and composition of AM fungi may differ from that of its core range.

To this day, 355 species of arbuscular mycorrhizal (AM) fungi are described within the Glomeromycota phylum (Větrovský et al., 2023). While it is generally accepted that AM fungi lack host specificity, our understanding of their functional traits, environmental preferences, and host associations remains incomplete. Sugar maple can associate with various AM fungi, and these partnerships can vary depending on environmental conditions. For example, Klironomos et al. (1993) showed that *Glomus* spp. were the most dominant and frequent colonizers of sugar maple roots in nature and were found in both alkaline and acidic soils, a finding supported by Coughlan et al. (2000). However, while species such as *G. borealis, G. melanospora*, and *Acaulospora* spp. showed a preference for acidic soils in Klironomos et al. (1993) and Coughlan et al. (2000) found that spore counts of *Acaulospora* spp. actually increased with higer pH. What's more, the authors found a preference of *Sclerocystis rubiformis* for low pH soils, with this taxon thought to be particularly competitive and well adapted in environments with low pH or with relatively elevated aluminum concentrations, highlighting the highly context-dependent nature of AM fungal associations (Coughlan et al., 2000). In more acidic podzolic soils, sugar maple can show high AM fungal colonization but with skewed vesicle-to-arbuscule ratios, indicating stress (Klironomos et al., 1993). With this, there is potential that the soil type and characteristics (e.g., pH, nutrients) impacts the function and efficiency of the AM-maple symbiosis, with subsequent cascading effects on host tree fitness. In any case, there is compelling evidence that AM fungi play a critical role in influencing sugar maple resilience to harsher conditions in podzolic and acidic soils (Coughlan et al., 2000; Klironomos et al., 1993; Ouimet et al., 1996), which are characteristic of northern range limits and higher elevations (even in an impaired symbiosis). This not only highlights the importance of regional species pools in fulfilling different needs for their hosts based on contrasting environmental conditions (on top of those conducive to their own life histories), but also in understanding the specific contexts behind which these associations form. While many AM fungi are presumed to have global distributions, their roles can differ significantly based on environmental conditions. This has important implications for the management of species that form these associations, particularly in consideration of shifting species ranges due to natural or assisted migration.

Tree ontogeny (i.e., host development) may be just as important to study in terms of expected associations between sugar maple and AM fungi. If we consider that different AM fungal groups can vary significantly in their functional and life history traits, then we should also consider how these different groupings may become more or less relevant symbionts throughout different stages of their host tree's development. As per the Competitor-Stress Tolerator-Ruderal framework (CSR theory; Grime, 1977) in relation to AM fungi (Chagnon et al., 2013), it is likely that different AM species' life history traits differentially align with specific host tree needs at various life cycle stages. Given the different challenges and stressors experienced by a tree during the transition from a seedling to a mature adult, it seems likely that the relative value of a certain suite of species and symbiotic traits would change over time. For example, as a seedling in a heavily competitive and volatile environment, the priority may be set toward ruderal symbionts that are fast growing, readily available, and thought to offer better protection from herbivores and pathogens (Chagnon et al., 2013; Delavaux et al., 2017) to which seedlings are particularly susceptible (Bayandala and Seiwa, 2016). As the tree matures, the priority could shift toward more “competitive” AM fungi that may act as larger, more efficient carbon sinks and build extensive hyphal networks for effective soil exploration and nutrient exploitation (Chagnon et al., 2013). These AM fungi may enhance water uptake and the supply of essential nutrients, such as phosphorus, which become increasingly important as the tree's biomass increases. Furthermore, and regardless of their stage of development, host plants may prefer to associate with more stress-tolerant AM fungi depending on local environmental factors (e.g., low pH; Klironomos, 1995). Such AM fungi may provide more benefits over time, including stability in nutrient exchange, compared to species that excel at carbon acquisition, but are less effective under stressful conditions or have shorter-lived mycelium (Chagnon et al., 2013). This would suggest that, despite a general lack of host specificity among AM fungi, certain fungal partners are more important under specific environmental conditions (Coughlan et al., 2000; Zahka et al., 1995). While this conceptual framework may be helpful in developing our understanding of sugar maple-AM interactions, it should be acknowledged that it takes a very plant-centric point of view and may not accurately reflect AM fungal traits and functions in different environments (Camenzind et al., 2024; Chaudhary et al., 2022). To address this, it is important to understand which AM fungal species and strains are found in different environmental contexts in association with sugar maple, how traits are expressed, and how AM-tree relationships may shift throughout tree ontogeny (Figure 3). Thus, outstanding questions include: (1) Which AM fungi thrive in which contexts and which life history traits favor this?; (2) Which AM fungal traits are most plastic and how does this affect their adaptability to changing environmental conditions?; and (3) How do AM symbioses change with tree ontogeny and ecological succession?

### 3.4 Microbe-microbe interactions in the hyphosphere

In contrast to the rhizosphere, the hyphosphere is the narrow zone of soil directly influenced by the exudation of labile carbon compounds via fungal hyphae. This area supports distinct microbial communities and associations that differ from those in the surrounding bulk soil (Wang et al., 2024). Similar to the rhizosphere, the hyphosphere is a zone of immense biological activity and interactions that are consequential for the plant-fungal symbiosis (Wang et al., 2024). Of particular interest are bacteria that are stimulated by hyphal exudates and transported along AM hyphae (Jiang et al., 2021). Interestingly, AM fungi appear to recruit specific bacterial taxa that fulfill certain ecological functions which they cannot perform themselves (Emmett et al., 2021; Wang et al., 2023). For example, in return for carbon compounds from mycorrhizal fungi, hyphosphere bacteria supply organic phosphorus by excreting phosphatase enzymes that solubilize phosphorous, rendering it available to the AM fungal partner (Jiang et al., 2021; Wang et al., 2024). Given that we still largely lack an understanding of the full suite of AM fungi that associate with sugar maple, it is not surprising that the hyphobiomes of these fungi have not been thoroughly considered or explored in the context of associations with sugar maple. However, an overlap of the key bacterial phyla involved in the processes driving the activity of hyphosphere (Wang et al., 2024, 2023) and those found in the roots of sugar maple do exist (e.g., Proteo- and Actinobacterial phyla) and warrant further exploration. By viewing the root endosphere, rhizosphere, and hyphosphere in their ensemble and as interacting habitats, we can hope to develop a truly comprehensive understanding of the multi-kingdom interactions taking place in the roots of sugar maple.

## 4 Phyllosphere

Similarly to belowground, the aboveground parts (the phyllosphere) of sugar maple trees host various communities of microorganisms, which inhabit branches, leaves, and flowers (De Bellis et al., 2022; Laforest-Lapointe et al., 2016a,b; Vujanovic and Brisson, 2002; Wallace et al., 2018). Microorganisms can live epiphytically (e.g., on the surface of plant compartments) and endophytically (e.g., inside and within cells of the leaves) on sugar maple (Demarquest and Lajoie, 2023; Vacher et al., 2016). There is accumulating evidence that phyllosphere bacteria and fungi residing on and within sugar maple leaves play important roles in their ecosystems, contributing to nutrient cycling, as well as influencing tree fitness, evolution, and plant community productivity (Laforest-Lapointe et al., 2017; Zilber-Rosenberg and Rosenberg, 2008). Yet, our knowledge of phyllosphere microorganisms, potential representatives of the core microbiome, and the role of microorganisms in sugar maple's response to global change is still limited.

### 4.1 Phyllosphere bacteria: key drivers and ecology

Bacteria are, as expected, prominent members of the sugar maple phyllosphere (De Bellis et al., 2022; Demarquest and Lajoie, 2023; Laforest-Lapointe et al., 2016a,b; Wallace et al., 2018). Several common bacterial taxa found in association with sugar maple include members of the classes Alpha- and Gamma-proteobacteria, Hymenobacteraceae, Beijerinckiaceae, Pseudo-monadaceae, and Methylobacteriaceae (Demarquest and Lajoie, 2023; Laforest-Lapointe et al., 2017, 2016a,b; Wallace et al., 2018). So far, research on bacterial communities associated with sugar maple has provided several key findings. For example, De Bellis et al. (2022) identified a diverse community of bacteria associated with sugar maple leaves and roots, highlighting their potential role in nutrient cycling and plant fitness. Then, Demarquest and Lajoie (2023) showed that leaf compartment primarily explained community diversity and composition variation, with epiphytic bacterial communities influenced by host and sites characteristics, while endophytic communities were more idiosyncratic. Together, these works highlighted the importance of priority effects and opportunistic/stochastic colonization in bacterial assembly. Laforest-Lapointe et al. (2017) examined the impact of urbanization, finding that urban environments significantly altered the composition and diversity of microbial communities, potentially affecting tree fitness, a pattern that was stronger for sugar and red maple species. Wallace et al. (2018) investigated interactions between sugar maple and endophytic bacteria, suggesting that certain bacterial strains could enhance tree growth and stress tolerance. Overall, these studies underscore the complexity and ecological significance of sugar maple phyllosphere bacterial communities, microbial responsiveness to environmental factors, and their potential implications for tree fitness.

Global change, including elevated temperatures, variation in relative humidity and soil moisture, as well as urbanization could significantly affect the bacterial communities colonizing the phyllosphere of sugar maple. For instance, recent studies have shown that beneficial microbes—such as bacteria that promote plant growth or assist sugar maple in coping with stress—may be negatively impacted by elevated temperatures and decreased relative humidity, ultimately reducing the trees' ability to adapt to changing conditions (Wemheuer et al., 2019; Xie et al., 2013). Urbanization factors, such as air pollution, can also affect the diversity, composition, and functioning of bacteria in the phyllosphere of trees. Notably, studies that investigated the effect of air pollution on bacterial communities showed that air pollution levels (e.g., PM_2.5_) had a stronger impact on bacterial diversity and composition than on fungi, with seasonal variations playing a significant role in shaping both bacterial and fungal communities (Fan et al., 2019). Air pollution can promote the growth of some bacterial species while suppressing others, thereby altering the overall structure of bacterial communities. Research on the effect of climate and urbanization on bacterial communities is further complicated by the presence of biofilms—aggregates of bacterial communities where cells adhere to one another and to surfaces, encased in a protective matrix of extracellular polymeric substances. For example, several studies showed that the mechanism of biofilm formation helps some phytopathogenic bacterial species (e.g., *Xanthomonas axonopodis* pv. citri*)*, to establish and spread disease on lemon tree leaves (Rigano et al., 2007), suggesting that this process may also favor the establishment of pathogenic bacteria. However, biofilms can also support bacterial communities that enhance the trees' ability to cope with environmental stressors. Investigating bacterial communities and biofilms in the phyllosphere of sugar maple is thus important for enhancing tree fitness and resilience to environmental stressors. While beneficial microbes can help trees adapt to climate change, the presence of pathogenic bacteria within biofilms presents potential risks that warrant careful consideration and management. In lieu of current knowledge gaps, the domain of tree-microbe interactions could benefit substantially from further studies on the impacts of urbanization and climate change on the phyllosphere microbes of sugar maple and related tree species.

### 4.2 Phyllosphere fungi: key drivers and ecology

Leaf-colonizing fungi display both beneficial and pathogen-like interactions with sugar maple and other maple species. On the positive side, a few foliar fungi can have beneficial effects on maple tree functioning, including protecting against pathogens (Xie et al., 2013), synthesizing growth hormones (Wemheuer et al., 2019), and providing nutrients (Huang et al., 2023). On the negative side, several genera of leaf fungi cause diseases in both sugar maple and other tree species (such as Norway maple), affecting trees in natural environments and urban areas (Lapointe and Brisson, 2011; Weiland and Stanosz, 2006). For several species of the maple genera, fungal diseases include leaf spot fungi such as *Septoria, Phyllosticta*, and *Didymosporina* which create unsightly spots (Horst, 2013); tar spots caused by *Rhydian acerinum, R. americanum*, and *R. punctatum* which form black patches and result in fall coloration and early leaf fall (Held et al., 2018); and finally anthracnose diseases from *Aureobasidium, Discula*, and *Kabatiella* spp. which lead to necrotic lesions often causing premature defoliation (Stanosz, 1993). Several biotrophic fungi (i.e., those that survive on living tissues exclusively), such as powdery mildew from *Erysiphe* and *Phyllactinia* spp., have also been shown to colonize Norway maple (Hudelson et al., 2008; Weiland and Stanosz, 2006). Yet, to our knowledge, there has been no record of these pathogens attacking sugar maple (so far). In addition to pathogens, fungal saprotrophs (e.g., fungi that degrade organic matter), may also reside on living leaves, while migrating belowground upon leaf senescence toward the end of the growing season (Liber et al., 2022; Sridhar and Bärlocher, 1992). All these fungal groups can be affected by tree genetic identities, age, individual physical, and chemical characteristics, as well as abiotic factors such as temperature, humidity, and sunlight exposure (Laforest-Lapointe et al., 2017; Vacher et al., 2016; Wallace et al., 2018). Overall, there is a notable lack of research on the ecology and life cycles of sugar maple beneficial and pathogenic fungi, with only a few exceptions, such as Myren et al. (1994). This is surprising given sugar maple's significant economic and cultural importance, keen scientific interest in understanding its decline, potential expansion of its range limits, along with predictions that soil-borne fungal pathogens will increase in abundance under projected climate change scenarios.

Sugar maple phyllosphere fungal communities can also be affected by climate change (Perreault and Laforest-Lapointe, 2022; Singh et al., 2023), an impact that was demonstrated for other temperate tree species, such as European beech (*Fagus sylvatica*; Cordier et al., 2012), English oak (*Quercus robur*; Faticov et al., 2021), and Balsam poplar (*Populus balsamifera*; Bálint et al., 2015). Rising temperatures, changing precipitation patterns, as well as higher atmospheric CO_2_, NO_2_, and fine particulate matter (PM_2.5_) can impact the composition and functions of fungi on leaves (Faticov et al., 2024; Huang et al., 2023). Shifts in temperature and humidity can alter fungal community dynamics, favoring heat-tolerant or drought-resistant species, while potentially reducing the diversity of beneficial microbes that play key roles in disease suppression and stress tolerance (Bálint et al., 2015; Faticov et al., 2021). For example, it was demonstrated that an overall rise in relative humidity may lead to a higher occurrence of fungal diseases in various plant genera (Romero et al., 2022). Temperature changes may also enhance the growth and spread of pathogenic fungi, increasing the likelihood of diseases such as anthracnose and tar spots (Singh et al., 2023). Fungal pathogens that have so far been absent in the sugar maple phyllosphere, such as powdery mildew species found on Norway maple, could colonize sugar maple under changing environmental conditions. Along with climatic factors, urbanization, and in particular air pollution, can either enhance or suppress fungal establishment and growth on trees (Cao et al., 2014; Fan et al., 2019). For example, Mcelrone et al. (2005) demonstrated that elevated CO_2_ significantly reduced disease incidence and severity of the fungal pathogen (*Phyllosticta minima*) on *A. rubrum* by decreasing stomatal conductance and altering leaf chemistry, despite enhanced fungal growth under higher CO_2_ levels. This warrants investigation into the impacts of air pollution on sugar maple fungal communities in urban environments. Overall, further research is needed to explore how abiotic factors and air pollution shape the diversity, composition, and functions of sugar maple phyllosphere fungi.

It is important to note that many of the aforementioned microbial groups, including fungi and bacteria, are also interacting with each other in ways that remain beyond our understanding (Chamard et al., 2024; Chaudhry et al., 2021). These interactions are also likely mediated by the host. In the case of sugar maple, there are very few studies that have investigated tree-fungi or tree-bacteria interactions in the phyllosphere (but see Demarquest and Lajoie, 2023; Laforest-Lapointe et al., 2016b,a; Wallace et al., 2018), not to mention studies that explore the interactions between microbial members, which is also largely true for other plant species. Therefore, further research is needed to explore (Figure 3): (1) Which microbes are interacting (with sugar maple and with each other)?; (2) How microbe-microbe interactions respond to climate-driven environmental changes?; and (3) How these interactions can influence sugar maple's response to novel climatic conditions?.

## 5 Conclusions and future directions

The interactions between sugar maple and its root- and leaf-associated microbial communities are fundamental to its fitness, growth, and adaptation. Yet, the current state of knowledge about the dynamics, functions, and roles that microbial communities play for sugar maple adaptation is limited. This review highlights the progress made in the last decades on microbial interactions in the rhizosphere and phyllosphere of sugar maple. Understanding the role of microbial communities for tree migration is essential, particularly as these microbes can influence nutrient acquisition, stress tolerance, and tree fitness. Below, we summarize several promising avenues that could bridge the current knowledge gaps.

### 5.1 Current limitations in studying sugar maple-microbe interactions

The current understanding of microbial communities associated with sugar maple relies mostly on 16S, ITS (internal transcribed spacer), and 18S rRNA short-read (~250bp) sequencing data. While marker gene sequencing has revolutionized our ability to characterize microbial diversity, it often provides identification with confidence only up to the genus level, with a notable lack of resolution at the species or strain level. This limitation is particularly pronounced for fungal communities, for which the simultaneous sequencing of ITS1 and ITS2 regions (~620bp) has been shown to provide the best taxonomic resolution (Ohta et al., 2023). It is further compounded by the fact that the concept of ‘species' varies significantly across life forms, with traditional biological or evolutionary species concepts often being inapplicable to microbes. Yet, many studies have revealed that host-microbe and microbe-microbe interactions occur at the level of intraspecific variants (Lloyd-Price et al., 2017). Consequently, many potentially critical interactions between microbial taxa and tree hosts remain unknown.

Moreover, molecular techniques can introduce primer-based biases due to preferential amplification of certain taxa over others, potentially leading to an underrepresentation of rare microbial groups (Abellan-Schneyder et al., 2021; Bellemain et al., 2010). This issue is problematic for fungi, as many species exhibit variable ribosomal RNA region (Nilsson et al., 2008). Furthermore, the existing reference databases are biased toward human microbiotas, especially bacteria, thus reducing our capacity to study polymicrobial environmental communities. As previously mentioned, the focus on bacteria and fungi has left a substantial gap in our knowledge of the roles of archaea, protists, and viruses that may also colonize sugar maple and interact with its bacterial and fungal communities. Another limitation arises from the lack of controlled experiments manipulating sugar maple microbiota in greenhouse or field settings. The potential resource allocation in extended fungal mycelium, which may play an important role in nutrient exchange and tree fitness, also remains largely a mystery. Lastly, very few sugar maple-associated bacterial and fungal taxa have been successfully cultured in laboratory settings or have their genomes fully sequenced. This emphasizes the need for the field to (1) leverage long-read or hybrid (shot- and long-read) sequencing strategies that would improve taxonomical resolution as well as reference databases; and (2) persevere in isolating microbial taxa from environmental samples to better characterize their identity and functions. In sum, the transition to more advanced molecular techniques such as transcriptomics and metabolomics will provide a more comprehensive understanding of sugar maple-microbe interactions.

Given the long-lived nature of sugar maple, it will also be necessary to further consider the temporal aspect of sugar maple-microbe interactions, particularly with tree ontogenic shifts. To date, most studies focusing on sugar maple rhizosphere and phyllosphere microbial communities have been done on tree seedlings or saplings, and typically over short time periods (<5 years). This focus represents an important bias, as it largely excludes mature trees, and should be addressed in future research. Microbial communities associated with mature sugar maples are likely to differ significantly from those found on seedlings, due to a range of factors, including differences in tree size, metabolic requirements, and the tree's influence on the stand and soil environment. When coupled with the differential impact climate change may have on younger trees vs. the mature individuals that constitute late-successional forests, it becomes even more crucial to understand the temporal dynamics of sugar maple-microbe interactions. Overall, long-term studies can help identify critical bottlenecks or stages in a tree's life where microbial community shifts may occur.

### 5.2 Microbes in the rhizosphere of sugar maple: challenges and future directions

In sugar maple's rhizosphere, the role of beneficial fungi such as AM in aiding nutrient acquisition and maintaining tree fitness is well-established, but several novel questions arise regarding their functionality (Figure 3). For example, how do these fungi interact with other microbial partners and contribute to nutrient cycling, and how are these interactions affected by soil chemistry and tree genetics? If sugar maple expands into new regions, alterations in symbiont availability could hinder seedling establishment, yet reduced herbivore and pathogen pressure might offset these effects. Conducting greenhouse experiments with soils and microbes from the center, edge, and beyond the predicted range, while also incorporating different sugar maple genotypes could provide valuable insights into the interactions and functions of microbes from different groups, allowing for a mechanistic characterization of their roles in tree fitness. Spatially and temporally resolved datasets of sugar maple-microbe interactions are required to develop accurate predictive models. These models could combine species distribution patterns and factors influencing sugar maple-microbe interactions in both current and predicted climatic conditions. Finally, reconciling the interconnected rhizosphere, root endosphere, and hyphosphere is imperative for understanding their joint impact on tree growth, establishment, and survival in the face of global change.

### 5.3 Outstanding questions

- Climate mismatches between tree range shifts and root microorganisms: are there any shifts in sugar maple-microbe interactions under climate change?
- How do sugar maple microbial communities vary across different environmental conditions, such as soil types, dominant tree species, temperature, and humidity? What are the dominant microbial taxa involved, and what specific functional roles do they play within the sugar maple rhizosphere?
- Which microbial taxa are critical for nitrogen fixation and phosphorus solubilization in the rhizosphere of sugar maple, and how do these functions vary with soil type?
- How do microbial communities shift during sugar maple ontogeny? How do these shifts influence nutrient acquisition and performance of sugar maple during different life stages?
- What is the role of AM fungi in sugar maple establishment in novel environments?
- What are the ecological roles (e.g., mutualist, pathogen, neutral) of DSE species?
- What are the functional roles and interactions of other symbiont groups, such as protists, archaea, and viruses, within the rhizosphere of sugar maple?

### 5.4 Microbes in the phyllosphere of sugar maple: challenges and future directions

Rising temperatures, urbanization, and changes in atmospheric conditions could alter the diversity and composition of phyllosphere microorganisms. For example, how do these shifts influence the ability of microbial communities to suppress pathogens, such as those causing anthracnose or tar spots, and promote stress tolerance? Furthermore, climate change could affect the virulence of pathogens, such as powdery mildew species currently associated with Norway maple, prompting research into whether new environmental pressures will alter the pathogenicity of these microorganisms as well as sugar maple susceptibility to new pathogens. Another important question is which role phyllosphere microorganisms play in assisting sugar maple migration northward. This includes understanding how variations in phyllosphere microbial diversity and composition might affect sugar maple fitness, nutrient uptake, and symbiotic interactions as it colonizes novel environments under climate change (Figure 3).

### 5.5 Outstanding questions

1. What are the mechanisms by which climate change affects the composition and functions of phyllosphere microorganisms in sugar maple?
2. How do microbial communities shift during tree development? Are there differences in succession patterns between phyllosphere and rhizosphere microbial communities during tree development?
3. How do microbial communities in the phyllosphere of sugar maple interact with each other, and what role does the host tree play in mediating these interactions?
4. What are the foliar pathogens of sugar maple; is there potential for a surge of novel pathogens as climate changes, and which factors contribute to their establishment?
5. What are the effects of urbanization on the assembly and functioning of leaf microbiomes, and what are the implications of these changes for tree fitness?
6. What role do biofilms play in the establishment and functioning of phyllosphere microbial communities, and is it influenced by environmental changes?
7. What role do phyllosphere microorganisms play in assisting sugar maple migration under climate change? How will shifts in microbial community diversity and community composition influence tree establishment in novel environments?
8. How do sugar maple belowground and aboveground microbial communities interact and influence each other in the context of global change?

## References

[B1] Abdelfattah A. Cacciola S. O. Mosca S. Zappia R. Schena L. (2017). Analysis of the fungal diversity in citrus leaves with greasy spot disease symptoms. Microb. Ecol. 73, 739–49. 10.1007/s00248-016-0874-x 27752718

[B2] Abdelfattah A. Tack A. J. M. Lobato C. Wassermann B. Berg G. (2023). From seed to seed: the role of microbial inheritance in the assembly of the plant microbiome. Trends Microbiol. 31, 346–55. 10.1016/j.tim.2022.10.009 36481186

[B3] Abdelfattah A. Wisniewski M. Schena L. Tack A. J. M. (2021). Experimental evidence of microbial inheritance in plants and transmission routes from seed to phyllosphere and root. Environ. Microbiol. 23, 2199–2214. 10.1111/1462-2920.15392 33427409

[B4] Abellan-Schneyder I. Matchado M. S. Reitmeier S. Sommer A. Sewald Z. Baumbach J.. (2021). Primer, pipelines, parameters: issues in 16S rRNA gene sequencing. mSphere 6, e01202–20. 10.1128/mSphere.01202-20 33627512 PMC8544895

[B5] Aitken S. N. Yeaman S. Holliday J. A. Wang T. Curtis-McLane S. (2008). Adaptation, migration or extirpation: climate change outcomes for tree populations. Evol. Appl. 1, 95–111. 10.1111/j.1752-4571.2007.00013.x 25567494 PMC3352395

[B6] Allsup C. M. George I. Lankau R. A. (2023). Shifting microbial communities can enhance tree tolerance to changing climates. Science. 380, 835–40. 10.1126/science.adf2027 37228219

[B7] Anthony M. A. Franz Bender S. van der Heijden M. (2023). Enumerating soil biodiversity. Proc. Nat. Acad. Sci. 120:e2304663120. 10.1073/pnas.2304663120 37549278 PMC10437432

[B8] Arnold W. Gewirtzman J. Raymond P. A. Duguid M. Brodersen M. Brown C.. (2024). A diverse and distinct microbiome inside living trees. bioRxiv. 10.1101/2024.05.30.596553 40770104

[B9] Asiegbu F. Kovalchuk A. (2021). Forest Microbiology, Volume 1: Tree Microbiome: Phyllosphere, Endosphere and Rhizosphere. London: Academic Press.

[B10] Averill C. Dietze M. C. Bhatnagar J. M. (2018). Continental-scale nitrogen pollution is shifting forest mycorrhizal associations and soil carbon stocks. Glob. Chang. Biol. 24, 4544–53. 10.1111/gcb.14368 30051940

[B11] Averill C. Werbin Z. R. Atherton K. F. Bhatnagar J. M. Dietze M. C. (2021). Soil microbiome predictability increases with spatial and taxonomic scale. Nat. Ecol. Evol. 5, 747–756. 10.1038/s41559-021-01445-9 33888877

[B12] Badri D. V. Quintana N. El Kassis E. Kim H. C. Hae Choi Y. Sugiyama A.. (2009). An ABC transporter mutation alters root exudation of phytochemicals that provoke an overhaul of natural soil microbiota. Plant Physiol. 151, 2006–17. 10.1104/pp.109.147462 19854857 PMC2785968

[B13] Bahram M. Netherway T. Hildebrand F. Pritsch K. Drenkhan R. Loit K.. (2020). Plant nutrient-acquisition strategies drive topsoil microbiome structure and function. New Phytol. 227, 1189–99. 10.1111/nph.16598 32279325

[B14] Baldrian P. (2017). Microbial activity and the dynamics of ecosystem processes in forest soils. Curr. Opin. Microbiol. Environm. Microbiol. 37, 128–34. 10.1016/j.mib.2017.06.008 28689057

[B15] Bálint M. Bartha L. OHara R. B. Olson M. S. Otte J. Pfenninger M. (2015). Relocation, high-latitude warming and host genetic identity shape the foliar fungal microbiome of poplars. Mol. Ecol. 24, 235–48. 10.1111/mec.13018 25443313

[B16] Bamisile B. S. Dash C. K. Akutse K. S. Keppanan R. Wang B. (2018). Fungal endophytes: beyond herbivore management. Front. Microbiol. 9:544. 10.3389/fmicb.2018.00544 29628919 PMC5876286

[B17] Barbour K. M. Barrón-Sandoval A. Walters K. E. Martiny J. B. H. (2023). Towards quantifying microbial dispersal in the environment. Environ. Microbiol. 25, 137–42. 10.1111/1462-2920.16270 36308707 PMC10100412

[B18] Bauce E. Allen D. C. (1992). Role of armillaria calvescens and glycobius speciosus in a sugar maple decline. Can. J. For. Res. 22, 549–52. 10.1139/x92-072

[B19] Bayandala Y. F. Seiwa K. (2016). Roles of pathogens on replacement of tree seedlings in heterogeneous light environments in a temperate forest: a reciprocal seed sowing experiment. J. Ecol. 104, 765–72. 10.1111/1365-2745.12552

[B20] Begum N. Qin C. Ashraf M. Zhang L. (2019). Role of arbuscular mycorrhizal fungi in plant growth regulation: implications in abiotic stress tolerance. Front. Plant Sci. 10:1068. 10.3389/fpls.2019.01068 31608075 PMC6761482

[B21] Bellemain E. Carlsen T. Brochmann C. Coissac E. Taberlet P. Kauserud H.. (2010). ITS as an environmental DNA barcode for fungi: an in silico approach reveals potential PCR biases. BMC Microbiol. 10:189. 10.1186/1471-2180-10-189 20618939 PMC2909996

[B22] Bennett J. A. Maherali H. Reinhart K. O. Lekberg Y. Hart M. M. Klironomos J.. (2017). Plant-soil feedbacks and mycorrhizal type influence temperate forest population dynamics. Science 355, 181–84. 10.1126/science.aai8212 28082590

[B23] Berendsen R. L. Pieterse C. M. J. Bakker P. A. H. M. (2012). The rhizosphere microbiome and plant health. Trends Plant Sci. 17, 478–86. 10.1016/j.tplants.2012.04.001 22564542

[B24] Boisvert-Marsh L. Pedlar J. H. de Blois S. Le Squin A. Lawrence K. McKenney D. W.. (2022). Migration-based simulations for canadian trees show limited tracking of suitable climate under climate change. Divers. Distribut. 28, 2330–48. 10.1111/ddi.13630

[B25] Bonfante A. Basile A. Bouma J. (2020). Targeting the soil quality and soil health concepts when aiming for the united nations sustainable development goals and the EU green deal. Soil 6, 453–66. 10.5194/soil-6-453-2020

[B26] Bose A. K. Weiskittel A. Wagner R. G. (2017). A three decade assessment of climate-associated changes in forest composition across the North-Eastern USA. J. Appl. Ecol. 54, 1592–1604. 10.1111/1365-2664.12917

[B27] Brice E. M. Miller B. A. Zhang H. Goldstein K. Zimmer S. N. Grosklos G. J.. (2020). Impacts of climate change on multiple use management of bureau of land management land in the Intermountain West, USA. Ecosphere 11:e03286. 10.1002/ecs2.3286

[B28] Brice M. H. Cazelles K. Legendre P. Josée Fortin M. (2019). Disturbances amplify tree community responses to climate change in the temperate–boreal ecotone. Global Ecol. Biogeogr. 28, 1668–81. 10.1111/geb.12971

[B29] Brown C. D. Vellend M. (2014). Non-climatic constraints on upper elevational plant range expansion under climate change. Proc. R. Soc. B Biol. Sci. 281:20141779. 10.1098/rspb.2014.1779 PMC4211457 25253462

[B30] Cahanovitc R. Livne-Luzon S. Angel R. Klein T. (2022). Ectomycorrhizal fungi mediate belowground carbon transfer between pines and oaks. ISME J. 16, 1420–29. 10.1038/s41396-022-01193-z 35042973 PMC9039061

[B31] Camenzind T. Haslwimmer H. Rillig M. C. Ruess L. Finn D. R. Tebbe C. C.. (2024). Revisiting soil fungal biomarkers and conversion factors: interspecific variability in phospholipid fatty acids, ergosterol and rDNA copy numbers. Soil Ecol. Lett. 6:240243. 10.1007/s42832-024-0243-5

[B32] Cao C. Jiang W. Wang B. Fang J. Lang J. Tian G.. (2014). Inhalable microorganisms in Beijings PM2.5 and PM10 pollutants during a severe smog event. Environm. Sci. Technol. 48, 1499–1507. 10.1021/es4048472 PMC3963435 24456276

[B33] Carteron A. Cichonski F. Laliberté E. (2022). Ectomycorrhizal stands accelerate decomposition to a greater extent than arbuscular mycorrhizal stands in a northern deciduous forest. Ecosystems 25, 1234–48. 10.1007/s10021-021-00712-x

[B34] Carteron A. Parasquive V. Blanchard F. Guilbeault-Mayers X. Turner B. L. Vellend M.. (2020). Soil abiotic and biotic properties constrain the establishment of a dominant temperate tree into boreal forests. J. Ecol. 108, 931–44. 10.1111/1365-2745.13326

[B35] Chagnon P. I. Bradley R. I. Maherali H. Klironomos J. N. (2013). A trait-based framework to understand life history of mycorrhizal fungi. Trends Plant Sci. 18:9. 10.1016/j.tplants.2013.05.001 23756036

[B36] Chamard J. Faticov M. Blanchet F. G. Chagnon P. L. Laforest-Lapointe I. (2024). Interplay of biotic and abiotic factors shapes tree seedling growth and root-associated microbial communities. Commun. Biol. 7:360. 10.1038/s42003-024-06042-7 38519711 PMC10960049

[B37] Chase J. M. Leibold M. A. (2003). Ecological Niches: Linking Classical and Contemporary Approaches. Chicago, IL: University of Chicago Press. Available at: https://press.uchicago.edu/ucp/books/book/chicago/E/bo3638660.html (accessed September 29, 2024).

[B38] Chaudhary V. B. Holland E. P. Charman-Anderson S. Guzman A. Bell-Dereske L. Cheeke T. E.. (2022). What are mycorrhizal traits? Trends Ecol. Evol. 37, 573–81. 10.1016/j.tree.2022.04.003 35504748

[B39] Chaudhary V. B. Nolimal S. Sosa-Hernández M. A. Egan C. Kastens J. (2020). Trait-based aerial dispersal of arbuscular mycorrhizal fungi. New Phytol. 228, 238–52. 10.1111/nph.16667 32421866

[B40] Chaudhry V. Runge P. Sengupta P. Doehlemann G. Parker J. E. Kemen E.. (2021). Shaping the leaf microbiota: plant–microbe–microbe interactions. J. Exp. Bot. 72, 36–56. 10.1093/jxb/eraa417 32910810 PMC8210630

[B41] Choreño-Parra E. M. Treseder K. K. (2024). Mycorrhizal fungi modify decomposition: a meta-analysis. New Phytol. 242, 2763–74. 10.1111/nph.19748 38605488

[B42] Choudoir M. J. DeAngelis K. M. (2022). A framework for integrating microbial dispersal modes into soil ecosystem ecology. iScience 25:103887. 10.1016/j.isci.2022.103887 35243247 PMC8866892

[B43] Clark F. E. (1949). “Soil microorganisms and plant roots,” in Advances in Agronomy, ed. A. G. Norman (San Diego, CA: Academic Press), 241–88.

[B44] Clements F. E. (1916). Plant Succession; an Analysis of the Development of Vegetation. Washington: Carnegie Institution of Washington.

[B45] Collin A. Messier C. Kembel S. W. Bélanger N. (2018). Can Sugar Maple Establish into the Boreal Forest? Insights from Seedlings under Various Canopies in Southern Quebec. Ecosphere 9:e02022. 10.1002/ecs2.2022

[B46] Connell J. H. (1971). “On the role of natural enemies in preventing competitive exclusion in some marine animals and in rain forest trees,” in Dynamics of Populations, eds. P. J. den Boer and G. R. Gradwell [Wageningen: Centre for Agricultural Publishing and Documentation (PUDOC)], 298–312.

[B47] Cooke J. C. Butler R. H. Madole G. (1993). Some observations on the vertical distribution of vesicular arbuscular mycorrhizae in roots of salt marsh grasses growing in saturated soils. Mycologia 85, 547–550.

[B48] Cooke M. A. Widden P. O'Halloran I. (1992). Morphology, Incidence and Fertilization Effects on the Vesicular-Arbuscular Mycorrhizae of *Acer saccharum* in a Quebec Hardwood Forest. Mycologia 84, 422–30. 10.1080/00275514.1992.12026156

[B49] Coolen S. van der Molen M. R. Welte C. U. (2022). The secret life of insect-associated microbes and how they shape insect–plant interactions. FEMS Microbiol. Ecol. 9:fiac083. 10.1093/femsec/fiac083 PMC9409087 35830517

[B50] Cordier T. Robin C. Capdevielle X. Fabreguettes O. Desprez-Loustau M. L. Vacher C.. (2012). The composition of phyllosphere fungal assemblages of European beech (*Fagus Sylvatica*) varies significantly along an elevation gradient. New Phytol. 196, 510–19. 10.1111/j.1469-8137.2012.04284.x 22934891

[B51] Cordovez V. Dini-Andreote F. Carrión V. J. Raaijmakers J. M. (2019). Ecology and evolution of plant microbiomes. Annu. Rev. Microbiol. 73, 69–88. 10.1146/annurev-micro-090817-062524 31091418

[B52] Coughlan A. P. Dalpé Y. Lapointe L. Piché Y. (2000). Soil pH-induced changes in root colonization, diversity, and reproduction of symbiotic arbuscular mycorrhizal fungi from healthy and declining maple forests. Canadian J. For. Res. 30, 1543–54. 10.1139/x00-090

[B53] Cross H. Sønstebø J. H. Nagy N. E. Timmermann V. Solheim H. Børja I.. (2017). Fungal diversity and seasonal succession in ash leaves infected by the invasive ascomycete hymenoscyphus fraxineus. New Phytol. 213, 1405–17. 10.1111/nph.14204 27716950 PMC5347882

[B54] Davis M. B. Shaw R. G. (2001). Range shifts and adaptive responses to quaternary climate change. Science 292, 673–79. 10.1126/science.292.5517.673 11326089

[B55] De Bellis T. Kembel S. W. Lessard J.-P. (2019). Shared mycorrhizae but distinct communities of other root-associated microbes on co-occurring native and invasive maples. PeerJ. 7:e7295. 10.7717/peerj.7295 31392089 PMC6677121

[B56] De Bellis T. Laforest-Lapointe I. Solarik K. A. Gravel D. Kembel S. W. (2022). Regional variation drives differences in microbial communities associated with sugar maple across a latitudinal range. Ecology 103:e3727. 10.1002/ecy.3727 35412652

[B57] De Bellis T. Widden P. Messier C. (2002). Effects of selective cuts on the mycorrhizae of regenerating betula alleghaniensis and *Acer saccharum* seedlings in two quebec mixed deciduous forests. Cana. J. Forest Res. 32, 1094–1102. 10.1139/x02-035

[B58] Delavaux C. S. LaManna J. A. Myers J. A. Phillips R P. Aguilar S. Allen D.. (2023). Mycorrhizal feedbacks influence global forest structure and diversity. Commun. Biol. 6, 1–11. 10.1038/s42003-023-05410-z 37857800 PMC10587352

[B59] Delavaux C. S. Smith-Ramesh L. M. Kuebbing S. E. (2017). Beyond nutrients: a meta-analysis of the diverse effects of arbuscular mycorrhizal fungi on plants and soils. Ecology 98, 2111–19. 10.1002/ecy.1892 28500779

[B60] Delisle-Houde M. Blais M. Tweddell R. J. Rioux D. (2021). Antibacterial activity of geraniin from sugar maple leaves: an ultrastructural study with the phytopathogen xanthomonas campestris Pv. vitians. J. Plant Pathol. 103, 461–71. 10.1007/s42161-021-00743-2 33551638 PMC7856855

[B61] Demarquest G. Lajoie G. (2023). Bacterial endophytes of sugar maple leaves vary more idiosyncratically than epiphytes across a large geographic area. FEMS Microbiol. Ecol. 99:fiad079. 10.1093/femsec/fiad079 37442613

[B62] Dickie I. A. Bufford J. L. Cobb R. C. Desprez-Loustau M. L. Grelet G. Hulme P. E.. (2017). The emerging science of linked plant–fungal invasions. New Phytol. 215, 1314–32. 10.1111/nph.14657 28649741

[B63] Eagar A. C. Smemo K. A. Phillips R. P. Blackwood C. B. (2023). Context-dependence of fungal community responses to dominant tree mycorrhizal types in northern hardwood forests. Soil Biol. Biochem. 178:108971. 10.1016/j.soilbio.2023.108971

[B64] Edwards C. A. Arancon N. Q. (2022). “Interactions between earthworms, microorganisms, and other invertebrates,” in Biology and Ecology of Earthworms, eds. C. A. Edwards and N. Q. Arancon (New York, NY: Springer US), 275–301.

[B65] Elton C. S. (1927). Animal Ecology. New York, Macmillan Co. Available at: http://archive.org/details/animalecology00elto (accessed November 9, 2020).

[B66] Emmett B. D. Lévesque-Tremblay V. Harrison M. J. (2021). Conserved and reproducible bacterial communities associate with extraradical hyphae of arbuscular mycorrhizal fungi. ISME J. 15, 2276–88. 10.1038/s41396-021-00920-2 33649552 PMC8319317

[B67] Fan X. Y. Gao J. F. Pan K. L. Li D. C. Li H. H. Dai H. H.. (2019). More obvious air pollution impacts on variations in bacteria than fungi and their co-occurrences with ammonia-oxidizing microorganisms in PM2.5′. Environm. Pollut. 251, 668–680. 10.1016/j.envpol.2019.05.004 31108300

[B68] Faticov M. Abdelfattah A. Hambäck P. Roslin T. Tack A. J. M. (2023). Different spatial structure of plant-associated fungal communities above- and belowground. Ecol. Evol. 13:e10065. 10.1002/ece3.10065 37223309 PMC10200691

[B69] Faticov M. Abdelfattah A. Roslin T. Vacher C. Hambäck P. Blanchet F. G.. (2021). Climate warming dominates over plant genotype in shaping the seasonal trajectory of foliar fungal communities on oak. New Phytol. 231, 1770–83. 10.1111/nph.17434 33960441

[B70] Faticov M. Amorim J. H. Abdelfattah A. van Dijk L. J. A. Carvalho A. C. Laforest-Lapointe I.. (2024). Local climate, air quality and leaf litter cover shape foliar fungal communities on an urban tree. Ambio 53, 1673–1685. 10.1007/s13280-024-02041-4 38871928 PMC11436615

[B71] Figueiredo A. F. Boy J. Guggenberger G. (2021). Common mycorrhizae network: a review of the theories and mechanisms behind underground interactions. Front. Fungal Biol. 2:735299. 10.3389/ffunb.2021.735299 37744156 PMC10512311

[B72] Filteau M. Lagacé L. LaPointe G. Roy D. (2010). Seasonal and regional diversity of maple sap microbiota revealed using community PCR fingerprinting and 16S rRNA gene clone libraries. Syst. Appl. Microbiol. 33, 165–73. 10.1016/j.syapm.2010.02.003 20202776

[B73] Forzieri G. Dakos V. McDowell N. G. Ramdane A. Cescatti A. (2022). Emerging signals of declining forest resilience under climate change. Nature 608, 534–39. 10.1038/s41586-022-04959-9 35831499 PMC9385496

[B74] Frank A. C. Guzmán J. P. S. Shay J. E. (2017). Transmission of bacterial endophytes. Microorganisms 5:70. 10.3390/microorganisms5040070 29125552 PMC5748579

[B75] Fryer J. L. (2018). “Tree species distribution maps from littles “atlas of united states trees” series,” in *Fire Effects Information System, [Online]. U.S. Department of Agriculture, Forest Service, Rocky Mountain Research Station, Fire Sciences Laboratory (Producer)*. Available at: https://www.fs.usda.gov/database/feis/pdfs/Little/aa_SupportingFiles/LittleMaps.html (accessed September 30, 2024).

[B76] Gao J. Liao P. C. Huang B. H. Yu T. Zhang Y. Y. Li J. Q. (2020). Historical biogeography of *Acer* L. (Sapindaceae): genetic evidence for out-of-asia hypothesis with multiple dispersals to North America and Europe. Scien. Rep. 10:21178. 10.1038/s41598-020-78145-0 PMC7712834 33273626

[B77] Gilliam F. S. (2016). Forest ecosystems of temperate climatic regions: from ancient use to climate change. New Phytol. 212, 871–87. 10.1111/nph.14255 27787948

[B78] Gleason H. A. (1939). The individualistic concept of the plant association. Am. Midl. Nat. 21, 92–110. 10.2307/2420377

[B79] Godman R. M. (1957). Silvical Characteristics of Sugar Maple (Acer Saccharum). Saint Paul, Minnesota: Lake States Forest Experiment Station, Forest Service, U.S. Department of Agriculture.

[B80] Gómez-Ruiz E. P. Lacher T. E. (2019). Climate change, range shifts, and the disruption of a pollinator-plant complex. Sci. Rep. 9:14048. 10.1038/s41598-019-50059-6 31575888 PMC6773846

[B81] Graignic N. Tremblay F. Bergeron Y. (2018). Influence of northern limit range on genetic diversity and structure in a widespread north american tree, sugar maple (*Acer saccharum* Marshall). Ecol. Evol. 8, 2766–80. 10.1002/ece3.3906 29531693 PMC5838051

[B82] Grime J. P. (1977). Evidence for the existence of three primary strategies in plants and its relevance to ecological and evolutionary theory. Am. Nat. 111, 1169–94. 10.1086/283244

[B83] Grinnell J. (1917). The niche-relationships of the California thrasher. AUK 34, 427–33. 10.2307/4072271

[B84] Grünig C. R. Queloz V. Sieber T. N. Holdenrieder O. (2008). Dark septate endophytes (DSE) of the phialocephala fortinii s.l. – Acephala applanata species complex in tree roots: classification, population biology, and ecology. Botany 86, 1355–69. 10.1139/B08-108

[B85] Hakimara M. Despland E. (2023). “Vertical stratification of leaf physical traits exerts bottom-up pressures on insect herbivory in a sugar maple temperate forest,” in Insect Conservation and Diversity (Chichester: John Wiley & Sons Ltd).

[B86] Harada M. Endo A. Wada S. Watanabe T. Epron D. Asakawa S.. (2024). Ubiquity of methanogenic archaea in the trunk of coniferous and broadleaved tree species in a mountain forest. Antonie Van Leeuwenhoek 117:107. 10.1007/s10482-024-02004-5 39060562

[B87] Hartmann H. Bastos A. Das A. J. Esquivel-Muelbert A. Hammond W. M. Martínez-Vilalta J.. (2022). Climate change risks to global forest health: emergence of unexpected events of elevated tree mortality worldwide. Annual Rev. Plant Biol. 73, 673–702. 10.1146/annurev-arplant-102820-012804 35231182

[B88] Hawkins H. J. Cargill R. I. M. Van Nuland M. E. Hagen S. C. Field K. J. Sheldrake M.. (2023). Mycorrhizal mycelium as a global carbon pool. Current Biol. 33, R560–73. 10.1016/j.cub.2023.02.027 37279689

[B89] Heděnec P. Nilsson L. O. Zheng H. Gundersen P. Schmidt I. K. Rousk J.. (2020). Mycorrhizal association of common european tree species shapes biomass and metabolic activity of bacterial and fungal communities in soil. Soil Biol. Biochem. 149:107933. 10.1016/j.soilbio.2020.107933

[B90] Held B. W. Hoffmeister D. Blanchette R. A. (2018). Occurrence of European tar spot (*Rhytisma acerinum*) on norway maple (*Acer platanoides*) causing severe infections in Minnesota. Plant Dis. 102, 2655–2655. 10.1094/PDIS-05-18-0816-PDN

[B91] Henriksson N. Marshall J. Högberg M. N. Högberg P. Polle A. Franklin O.. (2023). Re-examining the evidence for the mother tree hypothesis – resource sharing among trees via ectomycorrhizal networks. New Phytol. 239, 19–28. 10.1111/nph.18935 37149889

[B92] Hillel D. Hatfield J. L. (2005). Encyclopedia of Soils in the Environment. Amsterdam: Elsevier.

[B93] Horsley S. B. Long R. P. Bailey S. W. Hallett R. A. Wargo P. M. (2002). Health of Eastern North American sugar maple forests and factors affecting decline. North. J. Appl. Fores. 19, 34–44. 10.1093/njaf/19.1.34

[B94] Horst R. K. (2013). “Leaf spots,” in Westcotts Plant Disease Handbook, ed R. Kenneth Horst (Dordrecht: Springer Netherlands), 201–36.

[B95] Hoysted G. A. Field K. J. Sinanaj B. Bell C. A. Bidartondo M. I. Pressel S.. (2023). Direct nitrogen, phosphorus and carbon exchanges between mucoromycotina “fine root endophyte” fungi and a flowering plant in novel monoxenic cultures. New Phytol. 238, 70–79. 10.1111/nph.18630 36739554 PMC10952891

[B96] Hoysted G. A. Jacob A. S. Kowal J. Giesemann P. Bidartondo M. I. Duckett J. G.. (2019). Mucoromycotina fine root endophyte fungi form nutritional mutualisms with vascular plants1 [CC-BY]. Plant Physiol. 181, 565–77. 10.1104/pp.19.00729 31358684 PMC6776871

[B97] Huang S. Zha X. Fu G. (2023). Affecting factors of plant phyllosphere microbial community and their responses to climatic warming—a review. Plants 12:2891. 10.3390/plants12162891 37631103 PMC10458011

[B98] Hubbell S. P. (2001). The Unified Neutral Theory of Biodiversity and Biogeography. Princeton: Princeton University Press. Available at: https://press.princeton.edu/books/paperback/9780691021287/the-unified-neutral-theory-of-biodiversity-and-biogeography-mpb-32

[B99] Hudelson B. Smith D. Stanosz G. Hanson M. (2008). First report of sawadaea tulasnei powdery mildew of norway maple (*Acer platanoides*) in Wisconsin. Plant Dis. 92:485. 10.1094/PDIS-92-3-0485A 30769710

[B100] Hulshof C. M. Spasojevic M. J. (2020). The edaphic control of plant diversity. Global Ecol. Biogeogr. 29, 1634–50. 10.1111/geb.13151

[B101] Hutchinson G. E. (1959). Homage to santa rosalia or why are there so many kinds of animals? Am. Natural. 93, 145–59. 10.1086/282070

[B102] IPCC (2023). “Climate change 2023: synthesis report,” in Contribution of Working Groups I, II and III to the Sixth Assessment Report of the Intergovernmental Panel on Climate Change, eds. H. Lee and J. Romero (Geneva: IPCC).

[B103] Janzen D. H. (1970). Herbivores and the number of tree species in tropical forests. Am. Nat. 104, 501–28. 10.1086/282687

[B104] Jia S. Wang X. Yuan Z. Lin F. Ye J. Lin G.. (2020). Tree species traits affect which natural enemies drive the janzen-connell effect in a temperate forest. Nat. Commun. 11:286. 10.1038/s41467-019-14140-y 31941904 PMC6962457

[B105] Jiang F. Zhang L. Zhou J. George T. S. Feng G. (2021). Arbuscular mycorrhizal fungi enhance mineralisation of organic phosphorus by carrying bacteria along their extraradical hyphae. New Phytol. 230, 304–15. 10.1111/nph.17081 33205416

[B106] Jo I. Fei S. Oswalt C. M. Domke G. M. Phillips R. P. (2019). Shifts in dominant tree mycorrhizal associations in response to anthropogenic impacts. Sci. Adv. 5:eaav6358. 10.1126/sciadv.aav6358 30989116 PMC6457943

[B107] Jumpponen A. (2001). Dark septate endophytes – are they mycorrhizal? Mycorrhiza 11, 207–11. 10.1007/s005720100112

[B108] Kadowaki K. Yamamoto S. Sato H. Tanabe A. S. Hidaka A. Toju H.. (2018). Mycorrhizal fungi mediate the direction and strength of plant–soil feedbacks differently between arbuscular mycorrhizal and ectomycorrhizal communities. Commun. Biol. 1, 1–11. 10.1038/s42003-018-0201-9 30480098 PMC6244237

[B109] Karst J. Jones M. D. Hoeksema J. D. (2023). Positive citation bias and overinterpreted results lead to misinformation on common mycorrhizal networks in forests. Nat. Ecol. Evol. 7, 501–11. 10.1038/s41559-023-01986-1 36782032

[B110] Keane R. M. Crawley M. J. (2002). Exotic plant invasions and the enemy release hypothesis. Trends Ecol. Evol. 17, 164–70. 10.1016/S0169-5347(02)02499-0

[B111] Kellner J. R. Kendrick J. Sax D. F. (2023). High-velocity upward shifts in vegetation are ubiquitous in mountains of Western North America. PLOS Clim. 2:e0000071. 10.1371/journal.pclm.0000071

[B112] Khare E. Mishra J. Arora N. K. (2018). Multifaceted interactions between endophytes and plant: developments and prospects. Front. Microbiol. 9:02732. 10.3389/fmicb.2018.02732 PMC6249440 30498482

[B113] Klironomos J. N. (1995). Arbuscular mycorrhizae of *Acer saccharum* in different soil types. Can. J. Botany 73, 1824–30. 10.1139/b95-193

[B114] Klironomos J. N. Moutoglis P. Kendrick B. Widden P. (1993). A comparison of spatial heterogeneity of vesicular–arbuscular mycorrhizal fungi in two maple-forest soils. Can. J. Botany 71, 1472–80. 10.1139/b93-178

[B115] Ladwig L. M. Ratajczak Z. R. Ocheltree T. W. Hafich K. A. Churchill A. C. Frey S. J. K.. (2016). Beyond arctic and alpine: the influence of winter climate on temperate ecosystems. Ecology 97, 372–82. 10.1890/15-0153.1 27145612

[B116] Laforest-Lapointe I. Messier C. Kembel S. W. (2016a). Host species identity, site and time drive temperate tree phyllosphere bacterial community structure. Microbiome 4:27. 10.1186/s40168-016-0174-1 27316353 PMC4912770

[B117] Laforest-Lapointe I. Messier C. Kembel S. W. (2016b). Tree phyllosphere bacterial communities: exploring the magnitude of intra- and inter-individual variation among host species. PeerJ 4, e2367. 10.7717/peerj.2367 27635335 PMC5012278

[B118] Laforest-Lapointe I. Messier C. Kembel S. W. (2017). Tree leaf bacterial community structure and diversity differ along a gradient of urban intensity. mSystems 2, 10-1128. 10.1128/mSystems.00087-17 PMC5715107 29238751

[B119] Lana A. F. Thomas O. T. Peterson J. F. (1980). A virus isolated from sugar maple. Phytopathol. Z. 97.

[B120] Lapointe M. Brisson J. (2011). Tar spot disease on norway maple in north america: quantifying the impacts of a reunion between an invasive tree species and its adventive natural enemy in an urban forest. Écoscience 18, 63–69. 10.2980/18-1-3378

[B121] Laughlin D. C. Abella S. R. (2007). Abiotic and biotic factors explain independent gradients of plant community composition in ponderosa pine forests. Ecol. Modell. 205, 231–40. 10.1016/j.ecolmodel.2007.02.018

[B122] Leboeuf M. (2018). Paroles d'un bouleau jaune : tu n'es pas un individu, tu es un écosystème. Montréal, QC: Éditions MultiMondes.

[B123] Lee-Yaw J. A. McCune J. L. Pironon S. Sheth S. N. (2022). Species distribution models rarely predict the biology of real populations. Ecography 2022:e05877. 10.1111/ecog.05877

[B124] Li F. Zi H. Sonne C. Li X. (2023). Microbiome sustains forest ecosystem functions across hierarchical scales. Eco-Environment and Health 2, 24–31. 10.1016/j.eehl.2023.03.001 38074452 PMC10702926

[B125] Li J. Stukel M. Bussies P. Skinner K. Lemmon A. R. Lemmon E. M. Brown K.. (2019). Maple phylogeny and biogeography inferred from phylogenomic data. J. Syst. Evol. 57, 594–606. 10.1111/jse.12535

[B126] Liber J. A. Minier D. H. Hopkins A. S. Van Wyk J. Longley R. Bonito G. (2022). Maple and hickory leaf litter fungal communities reflect pre-senescent leaf communities. PeerJ 10:e12701. 10.7717/peerj.12701 35127279 PMC8801177

[B127] Lima J. S. Lenoir J. Hylander K. (2024). Potential migration pathways of broadleaved trees across the receding boreal biome under future climate change. Glob. Chang. Biol. 30, e17471. 10.1111/gcb.17471 39188066

[B128] Liu N. Jacquemyn H. Liu Q. Shao S. C. Ding G. Xing X.. (2022). Effects of a dark septate fungal endophyte on the growth and physiological response of seedlings to drought in an epiphytic orchid. Front. Microbiol. 13:961172. 10.3389/fmicb.2022.961172 35875551 PMC9304953

[B129] Lloyd-Price J. Mahurkar A. Rahnavard G. Crabtree J. Orvis J. Hall A. B.. (2017). Strains, functions and dynamics in the expanded human microbiome project. Nature 550, 61–66. 10.1038/nature23889 28953883 PMC5831082

[B130] López J. L. Fourie A. Poppeliers S. W. M. Pappas N. Sánchez-Gil J. J. de Jonge R.. (2023). Growth rate is a dominant factor predicting the rhizosphere effect. ISME J. 17, 1396–1405. 10.1038/s41396-023-01453-6 37322285 PMC10432406

[B131] Maisuria V. B. Hosseinidoust Z. Tufenkji N. (2015). Polyphenolic extract from maple syrup potentiates antibiotic susceptibility and reduces biofilm formation of pathogenic bacteria. Appl. Environ. Microbiol. 81, 3782–92. 10.1128/AEM.00239-15 25819960 PMC4421064

[B132] Mandyam K. G. Jumpponen A. (2014). Mutualism-parasitism paradigm synthesized from results of root-endophyte models. Front. Microbiol. 5:776. 10.3389/fmicb.2014.00776 25628615 PMC4290590

[B133] Marshall H. (1785). “Arbustrum americanum,” in Market-Street, Between Second and Third-Streets, ed. J. Crukshank.

[B134] Matthews S. N. Iverson L. R. (2017). Managing for delicious ecosystem service under climate change: can united states sugar maple (*Acer saccharum*) syrup production be maintained in a warming climate? Int. J. Biodiversity Sci. Ecosyst. Serv. Managem. 13, 40–52. 10.1080/21513732.2017.1285815

[B135] Mayerhofer M. S. Kernaghan G. Harper K. A. (2013). The effects of fungal root endophytes on plant growth: a meta-analysis. Mycorrhiza 23, 119–28. 10.1007/s00572-012-0456-9 22983627

[B136] Mcelrone A. J. Reid C. D. Hoye K. A. Hart E. Jackson R. B. (2005). Elevated CO2 reduces disease incidence and severity of a red maple fungal pathogen via changes in host physiology and leaf chemistry. Glob. Chang. Biol. 11, 1828–36. 10.1111/j.1365-2486.2005.001015.x

[B137] Mohanram S. Kumar P. (2019). Rhizosphere microbiome: revisiting the synergy of plant-microbe interactions. Ann. Microbiol. 69, 307–20. 10.1007/s13213-019-01448-9

[B138] Moran E. V. Thuiller W. Angert A. L. Garzón M. B. (2022). Editorial: predicting and managing climate-driven range shifts in plants. Front. Ecol. Evolut. 10:856213. 10.3389/fevo.2022.856213

[B139] Morin X. Viner D. Chuine I. (2008). Tree species range shifts at a continental scale: new predictive insights from a process-based model. J.Ecol. 96, 784–94. 10.1111/j.1365-2745.2008.01369.x

[B140] Morris M. Kushner P. J. Moore G. W. K. Mercan O. (2023). Atmospheric circulation patterns associated with extreme wind events in Canadian cities. J. Clim. 36, 4443–4461. 10.1175/JCLI-D-22-0719.1

[B141] Morrison D. J. Chu D. Johnson A. L. S. (1985). Species of armillaria in British Columbia. Can. J. Plant Pathol. 7, 242–46. 10.1080/07060668509501685

[B142] Murphy B. Chrétien A. Brown L. (2009). “How do we come to know? exploring maple syrup production and climate change in Near North Ontario,” in Geography. Available at: https://scholars.wlu.ca/brantford_gg/2 (accessed July 17, 2024).

[B143] Myren D. T. Laflamme G. Singh P. Magasi L. P. Lachance D. (1994). Tree Diseases of Eastern Canada. Ottawa: Canadian Forest Service, Headquarters, Science and Sustainable Development Directorate. Available at: https://ostrnrcan-dostrncan.canada.ca/handle/1845/236699 (accessed September 5, 2024).

[B144] Netherway T. Bengtsson J. Buegger F. Fritscher J. Oja J. Pritsch K.. (2024). Pervasive associations between dark septate endophytic fungi with tree root and soil microbiomes across Europe. Nat. Commun. 15:159. 10.1038/s41467-023-44172-4 38167673 PMC10761831

[B145] Netherway T. Bengtsson J. Krab E. J. Bahram M. (2021). Biotic interactions with mycorrhizal systems as extended nutrient acquisition strategies shaping forest soil communities and functions. Basic Appl. Ecol. 50, 25–42. 10.1016/j.baae.2020.10.002

[B146] Newman E. I. (1988). “Mycorrhizal links between plants: their functioning and ecological significance,” in Advances in Ecological Research, eds. M. Begon, A. H. Fitter, E. D. Ford, and A. Macfadyen (San Diego, CA: Academic Press), 243–70.

[B147] Newsham K. K. (2011). A meta-analysis of plant responses to dark septate root endophytes. New Phytol. 190, 783–93. 10.1111/j.1469-8137.2010.03611.x 21244432

[B148] N'guyen G. Q. Roblet C. Lagacé L. Filteau M. (2022). A metataxonomic analysis of maple sap microbial communities reveals new insights into maple syrup complexity. Front. Syst. Biol. 2:893007. 10.3389/fsysb.2022.893007

[B149] Nilsson R. H. Kristiansson E. Ryberg M. Hallenberg N. Larsson K. H. (2008). Intraspecific ITS variability in the kingdom fungi as expressed in the international sequence databases and its implications for molecular species identification. Evol. Bioinform. 4, EBO.S653. 10.4137/EBO.S653 PMC2614188 19204817

[B150] Ohta A. Nishi K. Hirota K. Matsuo Y. (2023). Using nanopore sequencing to identify fungi from clinical samples with high phylogenetic resolution. Sci. Rep. 13:9785. 10.1038/s41598-023-37016-0 37328565 PMC10275880

[B151] Orchard S. Hilton S. Bending G. D. Dickie I. A. Standish R. J. Gleeson D. B.. (2017). Fine endophytes (glomus tenue) are related to mucoromycotina, not glomeromycota. New Phytol. 213, 481–86. 10.1111/nph.14268 27768808

[B152] Ouimet R. Camiré C. Furlan V. (1996). Effect of soil K, Ca and Mg saturation and endomycorrhization on growth and nutrient uptake of sugar maple seedlings. Plant Soil 179, 207–16. 10.1007/BF00009330

[B153] Parmesan C. (2006). Ecological and evolutionary responses to recent climate change. Annual Rev. Ecol. Evol. Systemat. 37, 637–69. 10.1146/annurev.ecolsys.37.091305.110100

[B154] Pearman P. B. Guisan A. Broennimann O. Randin C. F. (2008). Niche dynamics in space and time. Trends Ecol. Evol. 23, 149–58. 10.1016/j.tree.2007.11.005 18289716

[B155] Pehl L. Butin H. (1994). “Endophytische Pilze in Blaettern von Laubbaeumen Und Ihre Beziehungen Zu Blattgallen (Zoocecidien),” in Mitteilungen Aus Der Biologischen Bundesanstalt Fuer Land-Und Forstwirtschaft, 297. Available at: https://agris.fao.org/search/en/providers/123819/records/647361ee53aa8c89630b5630 (accessed September 4, 2024).

[B156] Perreault R. Laforest-Lapointe I. (2022). Plant-microbe interactions in the phyllosphere: facing challenges of the anthropocene. ISME J. 16, 339–45. 10.1038/s41396-021-01109-3 34522008 PMC8776876

[B157] Pitel N. E. Yanai R. D. (2014). Abiotic and biotic factors influencing sugar maple health: soils, topography, climate, and defoliation. Soil Sci. Soc. Am. J. 78, 2061–70. 10.2136/sssaj2014.06.0240

[B158] Prescott C. E. Grayston S. J. (2023). TAMM review: continuous root forestry—living roots sustain the belowground ecosystem and soil carbon in managed forests. For. Ecol. Manage. 532:120848. 10.1016/j.foreco.2023.120848

[B159] Quiza L. Tremblay J. Pagé A. P. Greer C. W. Pozniak C. J. Li R.. (2023). The effect of wheat genotype on the microbiome is more evident in roots and varies through time. ISME Communications 3, 1–10. 10.1038/s43705-023-00238-4 37076737 PMC10115884

[B160] Raaijmakers J. M. Paulitz T. C. Steinberg C. Alabouvette C. Moënne-Loccoz Y. (2009). The rhizosphere: a playground and battlefield for soilborne pathogens and beneficial microorganisms. Plant Soil 321, 341–61. 10.1007/s11104-008-9568-6

[B161] Rauschendorfer J. Rooney R. Külheim C. (2022). Strategies to mitigate shifts in red oak (Quercus Sect. Lobatae) distribution under a changing climate. Tree Physiol. 42, 2383–2400. 10.1093/treephys/tpac090 35867476

[B162] Rigano L. A. Siciliano F. Enrique R. Sendín L. Filippone P. Torres P. S.. (2007). Biofilm formation, epiphytic fitness, and canker development in xanthomonas axonopodis Pv. Citri. MPMI 20, 1222–30. 10.1094/MPMI-20-10-1222 17918624

[B163] Robinson D. G. Ammer C. Polle A. Bauhus J. Aloni R. Annighöfer P.. (2024). Mother trees, altruistic fungi, and the perils of plant personification. Trends Plant Sci. 29, 20–31. 10.1016/j.tplants.2023.08.010 37735061

[B164] Romero F. Cazzato S. Walder F. Vogelgsang S. Bender S. F. van der Heijden M. G. A.. (2022). Humidity and high temperature are important for predicting fungal disease outbreaks worldwide. New Phytol. 234, 1553–56. 10.1111/nph.17340 33713447

[B165] Rousk J. Bååth E. Brookes P. C. Lauber C. L. Lozupone C. Caporaso J. G.. (2010). Soil bacterial and fungal communities across a pH gradient in an arable soil. ISME J. 4, 1340–51. 10.1038/ismej.2010.58 20445636

[B166] Rumbou A. Candresse T. von Bargen S. Büttner C. (2021). Next-generation sequencing reveals a novel emaravirus in diseased maple trees from a german urban forest. Front. Microbiol. 11:621179. 10.3389/fmicb.2020.621179 33488565 PMC7819872

[B167] Sangwan S. Prasanna R. (2022). Mycorrhizae helper bacteria: unlocking their potential as bioenhancers of plant-arbuscular mycorrhizal fungal associations. Microb. Ecol. 84, 1–10. 10.1007/s00248-021-01831-7 34417849

[B168] Santos M. Cesanelli I. Diánez F. Sánchez-Montesinos B. Moreno-Gavíra A. (2021). Advances in the role of dark septate endophytes in the plant resistance to abiotic and biotic stresses. J. Fungi 7:939. 10.3390/jof7110939 PMC8622582 34829226

[B169] Savage J. Vellend M. (2015). Elevational shifts, biotic homogenization and time lags in vegetation change during 40 years of climate warming. Ecography 38, 546–55. 10.1111/ecog.01131

[B170] Sieber T. N. (2007). Endophytic fungi in forest trees: are they mutualists? Fungal Biol. Rev. Fung. Endophytes, 21, 75–89. 10.1016/j.fbr.2007.05.004

[B171] Sieber T. N. Dorworth C. E. (1994). An ecological study about assemblages of endophytic fungi in *Acer macrophyllum* in British Columbia: in search of candidate mycoherbicides. Can. J. Botany 72, 1397–1402. 10.1139/b94-172

[B172] Singh B. K. Delgado-Baquerizo M. Egidi E. Guirado E. Leach J. E. Liu H.. (2023). Climate change impacts on plant pathogens, food security and paths forward. Nat. Rev. Microbiol. 2023, 1–17. 10.1038/s41579-023-00900-7 PMC10153038 37131070

[B173] Soka G. Ritchie M. (2014). Arbuscular mycorrhizal symbiosis and ecosystem processes: prospects for future research in tropical soils. Open J. Ecol. 04, 11–22. 10.4236/oje.2014.41002

[B174] Solarik K. A. Messier C. Ouimet R. Bergeron Y. Gravel D. (2018). Local adaptation of trees at the range margins impacts range shifts in the face of climate change. Global Ecol. Biogeog. 27, 1507–19. 10.1111/geb.12829

[B175] Spatafora J. W. Chang Y. Benny G. L. Lazarus K. Smith M. E. Berbee M. L.. (2016). A phylum-level phylogenetic classification of zygomycete fungi based on genome-scale data. Mycologia 108, 1028–46. 10.3852/16-042 27738200 PMC6078412

[B176] Sridhar K. R. Bärlocher F. (1992). Endophytic aquatic hyphomycetes of roots of spruce, birch and maple. Mycol. Res. 96, 305–8. 10.1016/S0953-7562(09)80942-8

[B177] Stanosz G. R. (1993). Symptoms, association, and pathogenicity of *discula campestris*, a cause of sugar maple seedling anthracnose. Plant Dis. 77:1022. 10.1094/PD-77-1022

[B178] Tedersoo L. Bahram M. Põlme S. Kõljalg U. Yorou N. S. Wijesundera R.. (2014). Global diversity and geography of soil fungi. Science 46:1256688. 10.1126/science.1256688 25430773

[B179] Tedersoo L. Bahram M. Zobel M. (2020). How mycorrhizal associations drive plant population and community biology. Science 367:eaba1223. 10.1126/science.aba1223 32079744

[B180] Teste F. P. Simard S. W. (2008). Mycorrhizal networks and distance from mature trees alter patterns of competition and facilitation in dry douglas-fir forests. Oecologia 158, 193–203. 10.1007/s00442-008-1136-5 18781333

[B181] Uprety Y. Asselin H. Bergeron Y. (2017). Preserving ecosystem services on indigenous territory through restoration and management of a cultural keystone species. Forests 8:194. 10.3390/f8060194

[B182] Urli M. Brown C. D. Perez R. N. Chagnon P. L. Vellend M. (2016). Increased seedling establishment via enemy release at the upper elevational range limit of sugar maple. Ecology 97, 3058–69. 10.1002/ecy.1566 27870043

[B183] Vacher C. Hampe A. Porté A. J. Sauer U. Compant S. Morris C. E.. (2016). The phyllosphere: microbial jungle at the plant–climate interface. Annu. Rev. Ecol. Evol. Syst. 47, 1–24. 10.1146/annurev-ecolsys-121415-032238

[B184] van der Heijden M. G. A. Bardgett R. D. van Straalen N. M. (2008). The unseen majority: soil microbes as drivers of plant diversity and productivity in terrestrial ecosystems. Ecol. Lett. 11, 296–310. 10.1111/j.1461-0248.2007.01139.x 18047587

[B185] van der Heijden M. G. A. Martin F. M. Selosse M. A. Sanders I. R. (2015). Mycorrhizal ecology and evolution: the past, the present, and the future. New Phytol. 205, 1406–23. 10.1111/nph.13288 25639293

[B186] Van Nuland M. E. Qin C. Pellitier P. T. Zhu K. Peay K. G. (2024). Climate mismatches with ectomycorrhizal fungi contribute to migration lag in north american tree range shifts. Proc. Nat. Acad. Sci. 121, e2308811121. 10.1073/pnas.2308811121 38805274 PMC11161776

[B187] Vandermeer J. H. (1972). Niche theory. Ann. Rev. Ecol. Evol. System. 3, 107–32. 10.1146/annurev.es.03.110172.000543

[B188] Vellend M. (2010). Conceptual synthesis in community ecology. Q. Rev. Biol. 85, 183–206. 10.1086/652373 20565040

[B189] Vellend M. Béhé M. Carteron A. Crofts A. L. Danneyrolles V. Gamhewa H. T.. (2021). Plant responses to climate change and an elevational gradient in Mont Mégantic National park, Québec, Canada. Northeast. Natural. 28, 4–28. 10.1656/045.028.s1102

[B190] Větrovský T. Kolaríková Z. Lepinay C. Hollá S. A. Davison J. Fleyberková A.. (2023). GlobalAMFungi: a global database of arbuscular mycorrhizal fungal occurrences from high-throughput sequencing metabarcoding studies. New Phytol. 240, 2151–63. 10.1111/nph.19283 37781910

[B191] Vitasse Y. Ursenbacher S. Klein G. Bohnenstengel T. Chittaro Y. Delestrade A.. (2021). Phenological and elevational shifts of plants, animals and fungi under climate change in the European alps. Biological Reviews 96, 1816–35. 10.1111/brv.12727 33908168

[B192] Vujanovic V. Brisson J. (2002). A comparative study of endophytic mycobiota in leaves of *Acer saccharum* in Eastern North America. Mycol. Prog. 1, 147–54. 10.1007/s11557-006-0014-y

[B193] Walder F. Niemann H. Natarajan M. Lehmann M. F. Boller T. Wiemken A.. (2012). Mycorrhizal networks: common goods of plants shared under unequal terms of Trade1[W][OA]. Plant Physiol. 159, 789–97. 10.1104/pp.112.195727 22517410 PMC3375941

[B194] Wallace J. Laforest-Lapointe I. Kembel S. W. (2018). Variation in the leaf and root microbiome of sugar maple (*Acer saccharum*) at an elevational range limit. PeerJ. 6:e5293. 10.7717/peerj.5293 30128178 PMC6097496

[B195] Wang K. Wen Z. Asiegbu F. O. (2022). The dark septate endophyte phialocephala sphaeroides suppresses conifer pathogen transcripts and promotes root growth of norway spruce. Tree Physiol. 42, 2627–39. 10.1093/treephys/tpac089 35878416 PMC9743008

[B196] Wang L. George T. S. Feng G. (2024). Concepts and consequences of the hyphosphere core microbiome for arbuscular mycorrhizal fungal fitness and function. New Phytol. 242, 1529–33. 10.1111/nph.19396 38044555

[B197] Wang L. Zhang L. George T. S. Feng G. (2023). A core microbiome in the hyphosphere of arbuscular mycorrhizal fungi has functional significance in organic phosphorus mineralization. New Phytol. 238, 859–73. 10.1111/nph.18642 36444521

[B198] War A. F. Bashir I. Reshi Z. A. Kardol P. Rashid I. (2023). Insights into the seed microbiome and its ecological significance in plant life. Microbiol. Res. 269:127318. 10.1016/j.micres.2023.127318 36753851

[B199] Watkinson S. C. Boddy L. Money N. P. (2015). The Fungi, 3rd Edn. Cambridge, MA: Academic Press.

[B200] Weiland J. Stanosz G. (2006). Sawadaea tulasnei powdery mildew of norway maple (*Acer platanoides*) in North America. Plant Dis. 90:830. 10.1094/PD-90-0830C 30781261

[B201] Wemheuer F. Wemheuer B. Daniel R. Vidal S. (2019). Deciphering bacterial and fungal endophyte communities in leaves of two maple trees with Green Islands. Sci. Rep. 9:14183. 10.1038/s41598-019-50540-2 31578453 PMC6775154

[B202] Wisz M. S. Pottier J. Kissling W. D. Pellissier L. Lenoir J. Damgaard C. F.. (2013). The role of biotic interactions in shaping distributions and realised assemblages of species: implications for species distribution modelling. Biol. Rev. Camb. Philos. Soc. 88, 15–30. 10.1111/j.1469-185X.2012.00235.x 22686347 PMC3561684

[B203] Wolfe J. A. Tanai T. (1987). Systematics, phylogeny, and distribution of acer (maples) in the cenozoic of western north america. J. Faculty Sci. 22, 1–246.

[B204] Xiang Q. Y. Zhang W. H. Ricklefs R. E. Qian H. Chen Z. D. Wen J.. (2004). Regional differences in rates of plant speciation and molecularevolution: a comparison between Eastern Asia and Eastern North America. Evolution 58, 2175–84. 10.1111/j.0014-3820.2004.tb01596.x 15568220

[B205] Xie J. Strobel G. A. Mends M. T. Hilmer J. Nigg J. Geary B.. (2013). Collophora aceris, a novel antimycotic producing endophyte associated with douglas maple. Microb. Ecol. 66, 784–95. 10.1007/s00248-013-0281-5 23996143

[B206] Xu W. Prescott C. E. (2024). Can assisted migration mitigate climate-change impacts on forests? For. Ecol. Manage. 556:121738. 10.1016/j.foreco.2024.121738

[B207] Zahka G. A. Baggett K. L. Wong B. L. (1995). Inoculum potential and other vam fungi parameters in four sugar maple forests with different levels of stand dieback. For. Ecol. Manage. 75, 123–34. 10.1016/0378-1127(95)03536-J

[B208] Zilber-Rosenberg I. Rosenberg E. (2008). Role of microorganisms in the evolution of animals and plants: the hologenome theory of evolution. FEMS Microbiol. Rev. 32, 723–35. 10.1111/j.1574-6976.2008.00123.x 18549407

